# Atomically thin photoanode of InSe/graphene heterostructure

**DOI:** 10.1038/s41467-020-20341-7

**Published:** 2021-01-04

**Authors:** Haihong Zheng, Yizhen Lu, Kai-Hang Ye, Jinyuan Hu, Shuai Liu, Jiawei Yan, Yu Ye, Yuxi Guo, Zhan Lin, Jun Cheng, Yang Cao

**Affiliations:** 1 State Key Laboratory of Physical Chemistry of Solid Surfaces, Collaborative Innovation Center of Chemistry for Energy Materials (iChEM), College of Chemistry and Chemical Engineering, Xiamen University, Xiamen, 361005 China; 2 Guangzhou Key Laboratory of Clean Transportation Energy Chemistry, School of Chemical Engineering and Light Industry, Guangdong University of Technology, Guangzhou, 510006 China; 3 State Key Laboratory for Mesoscopic Physics, School of Physics, Peking University, Beijing, 100871 China; 4 Pen-Tung Sah Institute of Micro-Nano Science and Technology, Xiamen University, Xiamen, 361005 China

**Keywords:** Photocatalysis, Electrochemistry, Two-dimensional materials

## Abstract

Achieving high-efficiency photoelectrochemical water splitting requires a better understanding of ion kinetics, e.g., diffusion, adsorption and reactions, near the photoelectrode’s surface. However, with macroscopic three-dimensional electrodes, it is often difficult to disentangle the contributions of surface effects to the total photocurrent from that of various factors in the bulk. Here, we report a photoanode made from a InSe crystal monolayer that is encapsulated with monolayer graphene to ensure high stability. We choose InSe among other photoresponsive two-dimensional (2D) materials because of its unique properties of high mobility and strongly suppressing electron–hole pair recombination. Using the atomically thin electrodes, we obtained a photocurrent with a density >10 mA cm^−2^ at 1.23 V versus reversible hydrogen electrode, which is several orders of magnitude greater than other 2D photoelectrodes. In addition to the outstanding characteristics of InSe, we attribute the enhanced photocurrent to the strong coupling between the hydroxide ions and photo-generated holes near the anode surface. As a result, a persistent current even after illumination ceased was also observed due to the presence of ions trapped holes with suppressed electron-hole recombination. Our results provide atomically thin materials as a platform for investigating ion kinetics at the electrode surface and shed light on developing next-generation photoelectrodes with high efficiency.

## Abstract (web summary)

Achieving efficient photoelectrochemical cells requires insights into the ion kinetics at the surface of photoelectrode. Here, the authors show a new platform for investigating strong coupling between hydroxide ions and surface charge carriers by using monolayer InSe as the anode with high photocurrent density.

## Introduction

In photoelectrochemical (PEC) water splitting, the oxygen evolution reaction (OER) is considered to be the rate-limiting step compares to its hydrogen evolution counterpart reaction. This is because OER has four electron transferring processes along with complexities in the coupled ion and photogenerated electron/hole transport mechanism near the photoanode surface^1–5^. As a result, a better understanding of the interactions between ions in the electric double layer and charge carriers in the few atomic layers at the anode surface is critical for developing efficient PEC cells^6–8^. However, despite strong interest, experiments purely investigating the surface process have been proven challenging. The presence of various effects in the bulk electrode, including charge carriers’ short diffusion length and bulk recombination, suppress or obscure the efficiency of the surface chemical reactions^7,9^. Nevertheless, strategies to reduce electrode thickness down to tens of nanometers, which is smaller than the hole diffusion length, have been reported with anodes demonstrating enhanced PEC current^10,11^.

Most recently, two-dimensional (2D) crystals have been introduced as effective photoelectrodes materials in PEC reactions with improved photocatalytic properties. Such 2D materials have been demonstrated to have a direct band gap in the visible range, strong light absorption efficiency, and unique light-matter interactions^12,13^. Importantly, the possibility of assembling various 2D crystals into heterostructures offers another degree of freedom to adjust the band structure of these materials^14–17^. The resulting devices have been demonstrated to have tunable band gaps and elongated photo electron-hole pair lifetimes, which properties are essential for high-efficiency PEC water splitting^18–20^.

Among all 2D materials, InSe has become a promising candidate for PEC photoanodes due to its excellent charge carrier mobility and its reduced photogenerated electron-hole recombination rate^21^. The valence band edge of InSe is more positive than the oxidative potential of water. Its narrow band gap of ~1.2 eV further facilitates sunlight absorption across broad wavelengths^22,23^. The possibility of reducing InSe crystal thickness down to an atomic-scale makes it a reliable model system for studying surface ion diffusion and adsorption behavior with no influence from its bulk counterparts. However, thin InSe films are sensitive to water and oxygen and are chemically unstable at ambient conditions, which makes using it as an electrochemical electrode challenging^24^.

**Fig. 1:**
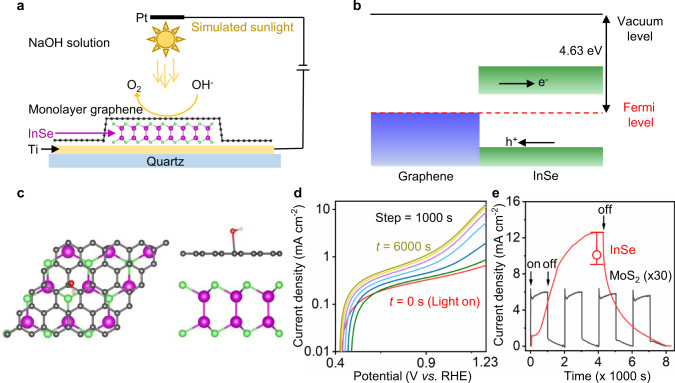
InSe/Gr anode for PEC measurements. **a** Schematic of our experiment setup. The illumination used in this report is simulated sunlight: AM 1.5 G one-sun (100 mW cm^−2^). **b** Band structure of InSe/Gr heterostructure. **c** DFT simulation of hydroxide species adsorption on InSe/Gr heterostructure surface. Left is the top view. Right is the side view. Red, white, gray, purple, and green balls represent oxygen, hydrogen, carbon, indium and selenium atoms, respectively. The hydroxide species adsorbs on top of the graphene carbon atom. **d** Linear sweep voltammetry of a representative monolayer InSe/Gr device measured at different illumination times. The original linear-scale curves are shown in Supplementary Fig. 5. The Ti electrodes and graphene top layer have little (<1%) contribution to the measured total photocurrent (Supplementary Fig. 6). **e** Photocurrent density of the PEC cell as a function of the time when light was on or off. In this report, all the *J*–*t* curves are measured at a potential of 1.23 V vs. RHE. The blank dot is the saturated maximum current density averaged from 4 samples. The error bar represents the standard deviation error.

With this in mind, in this work, we present a new class of PEC anodes made from single-crystal InSe encapsulated by a monolayer graphene, as shown in the schematics in Fig. 1a. The unique feature detailed herein is the atomic-scale thickness of the 2D InSe/graphene (InSe/Gr) photoelectrodes, allowing us to investigate the surface effects on PEC performance. The encapsulation by graphene offers a reliable method to enhance the stability of a monolayer InSe anode over an extended PEC cell operation life. Using the 2D heterostructure-based photoanode, we observed a photocurrent density >10 mA cm^−2^ at 1.23 V vs. the reversible hydrogen electrode (RHE). The detailed investigation of both experiments and DFT simulations reveal the importance of hydroxide ion interaction with trapped photogenerated holes near the anode surface. As a result, we also found a persistent current even after illumination ceased when using the thin InSe/graphene photoanode layers, which has not been observed in other 2D photoelectrodes.

## Results

### Fabrication of the InSe/Gr anode

The fabrication of InSe photoelectrodes is described in the “Methods” section. In brief, thin InSe crystals and monolayer graphene were obtained by mechanical exfoliation and then being transferred onto Ti electrodes on a quartz substrate. The unique feature detailed herein is the construction of InSe/graphene van der Waals (vdW) vertical heterostructures (see Supplementary Fig. 1 for an image of a representative InSe/Gr heterostructure and the atomic force microscope (AFM) characterization of the flake thicknesses). In this structure, the chemically less stable InSe crystals were encapsulated by graphene. All the fabrication procedures were performed in a glove box filled with an inert gas (argon) atmosphere to avoid InSe degradation^25^. This encapsulation effectively enabled the high stability of the resulting photoanodes with an InSe thickness down to a monolayer. Atomically thin graphene with optical transparency and high conductivity further facilitates the light absorption and charge carrier transport between InSe and ions in the PEC solution^13,19^.

### PEC measurements

The importance of building a InSe/Gr heterostructure can be better understood by analyzing hydroxide ions (OH^−^) and photo-generated hole migration near the anode surface using density functional theory (DFT) simulations (Supplementary Methods ‘DFT analysis’; Supplementary Figs. 2–4; Supplementary Tables 1–4). As shown in Fig. 1b, the InSe/Gr heterostructure was calculated to have a chemical potential of ~−4.6 eV (vs. vacuum), which is well above the OER level of −4.9 eV at pH = 13. The band alignment between InSe and graphene generates a built-in electric field and, in turn, drives holes to the surface of the electrode. In addition, the InSe/Gr heterostructure enables OH^−^ accumulation near the anode surface, with a calculated adsorption energy ~−0.74 eV (Fig. 1c). Indeed, a preferential adsorption of hydroxide ions at carbon surfaces has been reported, with a similar adsorption energy on a graphene surface^26,27^. As a result, both negatively charged OH^−^ species and photo-generated holes are enriched near the InSe/Gr anode surface, facilitating their possible interaction.

The current-voltage characteristics of the devices using monolayer InSe were measured in 0.2 M NaOH solutions and are summarized in Fig. 1d, e. With simulated sunlight illumination, the recorded linear sweep voltammetry (LSV) shows a hydroxide ion oxidation onset potential of ~420 mV. The measured photocurrent density *J* was found to increase as a function of the illumination time *t*, and this yielded a large saturation current density *J*_max_ > 10 mA cm^−2^ at 1.23 V vs. RHE. This value is at least two-fold greater than that of the state-of-art photoanodes, such as BiVO_4_, which has been demonstrated to have the highest photocurrent density (usually <5 mA cm^−2^, albeit when decorated with various co-catalyst) among others^28,29^. Moreover, the anode reaction persists even after the illumination ceases, with a typical decay time *t*_D_ of ~4000 s before the observed *J* decays from *J*_max_ to the dark current level. In a typical *J*–*t* cycle time of 8000 s, as shown in Fig. 1e, the persistent *J* contributes ~20% to the total number of oxidized hydroxide ions and, thus, further enhances the efficiency of the solar energy conversion. There is no obvious degradation of our devices and little variation (<5%) in their *J*_max_ within a typical lifetime of one month, which confirms the stability of InSe crystals with graphene encapsulation (Supplementary Fig. 7). The importance of the 2D InSe anode to PEC efficiency was further confirmed by the following control experiments. We measured monolayer MoS_2_/graphene devices using the same conditions, as shown in Fig. 1e and Supplementary Fig. 8. While there are similarities between InSe and MoS_2_ (e.g., atomically thin, suitable band diagram for OER and relatively high carrier mobility), the MoS_2_ shows a fast *J* photo response with a smaller *J*_max_. Such behavior is in consistent with MoS_2_ anodes previously reported^30,31^.

### Hydroxide ion adsorption on the InSe/Gr surface

**Fig. 2:**
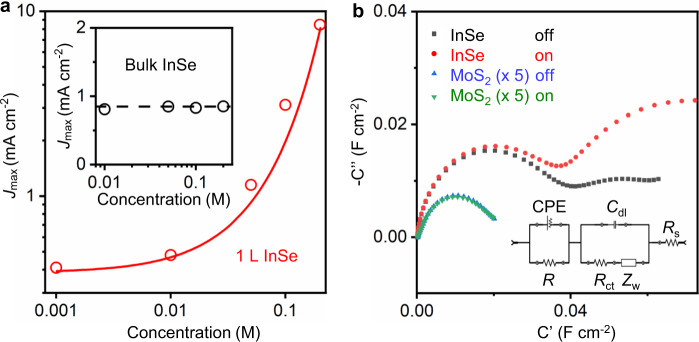
Hydroxide ion adsorption on the InSe/Gr surface. **a** *J*_max_ of a InSe/Gr monolayer device as a function of the NaOH concentration. Red dots are the experimental data. The red line is the prediction using Eq. (1) with a surface charge density of 1 mC m^−2^. The inset is *J*_max_ of the bulk (thickness ~1 μm) InSe/Gr device as a function of the NaOH concentration. The black dots are the experimental data. The black dashed line is to guide the eye. **b** Electrochemical impedance spectroscopy of InSe/Gr and MoS_2_/Gr monolayer devices when the light is on and off. The inset shows an equivalent electrical circuit of the InSe/Gr anode electrochemical interface. The first semicircle close to origin represents the interface capacitance between the InSe/Gr and the Ti constant phase element (CPE), and the second semicircle represents the interface capacitance between the InSe/Gr and the PEC solution. For additional details, see Supplementary Methods, ‘EIS’ section.

To gain better insight into the surface effects on the unique performance of the InSe/Gr anode, we first investigated *J*_max_ as a function of the NaOH bulk concentration *C* (Supplementary Fig. 9). As shown in Fig. 2a, *J*_max_ scales proportionally at high *C* but deviates from the linear dependence and saturates in the limit of small *C*. Such observation is a signature of ion kinetics caused by surface charges, the density of which is independent of the salt concentration^32,33^. Accordingly, the results in Fig. 2a indicate that the OH^−^ concentration near the anode surface *C*_surf_ is dominated by both the surface charge density *ρ* and the bulk concentration *C*. In the low-*C* region, the constant *J*_max_ suggests a constant number of ions adsorbed on surface. The saturation behavior vanishes in the high-*C* region where the number of ions close to surface is dominated by the bulk concentration. Thus, we quantitatively analyzed *ρ*:

1 $$J_{\max } \propto C_{{\mathrm{surf}}} = C_{{\mathrm{ad}}} + C = \frac{\rho }{{{\mathrm{F}}d}} + C$$

where *C*_ad_ is the concentration contribution from OH^−^ adsorbed on the surface, F is the Faraday constant, *d* is the Stern layer thickness, and *d* is ~1 nm. Best fitting using Eq. (1) to Fig. 2a yields *ρ* ~ 1 mC m^−2^, which is one order of magnitude smaller than the value recently reported for carbon materials (~10 mC m^−2^)^34^. We believe that the low surface charge density in our electrodes is due to their clean surface condition. The mechanically exfoliated top graphene contains very few, if any, surface defects^35,36^. Eq. (1) also suggests that at low *C*, hydroxide ions accumulate near the electrode surface with *C*_ad_ being significantly greater than *C*, up to 10 times at *C* = 0.001 M in our case.

It is illustrative to estimate the photo-generated hole concentration *C*_hole_ in the InSe anode and to compare that value with *C*_surf_, since *J*_max_ is expected to be limited by the lower concentration of these two values. After illumination ceases, the measured persistent current comes from OH^−^ oxidation with holes. Thus, we calculated *C*_hole_ = 1/e *∫ J*d*t*, where e is the electric charge. The integral is between *t* when illumination ceases and when *J* decays to that of the dark state. In monolayer InSe, the above equation yields *C*_hole_ > 3 × 10^19^ cm^−2^ (or 5 × 10^5^ M, with a InSe thickness ~1 nm), which is several orders of magnitude greater than *C*_surf_. This indicates the limiting concentration for *J*_max_ is *C*_surf_, confirming the accuracy of our analysis in Fig. 2a. We note that the estimated *C*_hole_ is order of magnitudes greater than that of InSe photoelectric devices measured in vacuum and at ambient conditions^21^. Thus, the surface adsorbed OH^−^ species are hole traps that lead to concentrated holes in the InSe/Gr electrodes. Together with Fig. 2b, where we demonstrate the presence of the hole-enriched surface OH^−^, our results suggest a strong coupling between the OH^−^ and the photo-generated holes.

The observed surface ion adsorption was further confirmed using electrochemical impedance spectroscopy (EIS). The EIS measurements show that under illumination, the capacitance at the interface of InSe/Gr and the solution is ~2.3 times greater than that when illumination is off (Fig. 2b and Supplementary Table 5). This result suggests an interaction between the photo-generated holes and OH^−^ species that further enriches the concentration of the latter^37^. This result also matches our DFT analysis of the InSe/Gr band structure. As a reference, the capacitance at the MoS_2_/Gr electrode surface has little dependence on the illumination, with its absolute value being 82 times less than that of InSe/Gr electrodes under illumination (Supplementary Table 5). The smaller capacitance indicates a less favored OH^−^ surface adsorption and is qualitatively in agreement with the lower *J*_max_ from MoS_2_/Gr observed in Fig. 1c.

### The persistent current after illumination ceases

**Fig. 3:**
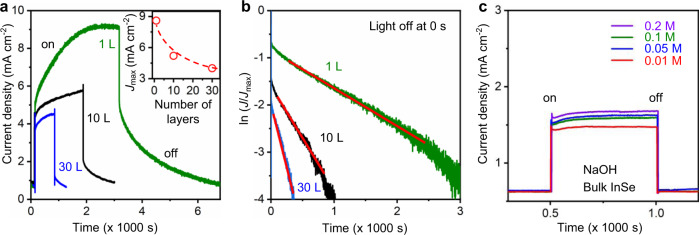
Persistent photocurrent density in the InSe/Gr photoanode. **a** Current density as a function of illumination time for InSe with various thicknesses. Inset shows the observed *J*_max_ as a function of the InSe thickness. **b** Photocurrent decay for InSe with various thicknesses. The time point at which the light ceased is defined as *t* = 0 for each sample. The red lines are the best fit using Eq. (2). **c** Temporal dependence of the bulk InSe/Gr anode with various NaOH concentrations.

Having established the OH^−^-hole interaction governed by the high photocurrent density, we next explore the mechanism behind the persistent *J* on InSe/Gr anodes after illumination ceases. We measured the photocurrent response in InSe flakes with various thicknesses, which provides information on the surface effects of the InSe/Gr anode. To minimize the contribution of surface charge-induced hydroxide ion accumulation, we used NaOH solutions having a relatively high concentration of 0.5 M. The *J*–*t* characteristics in Fig. 3a show that with increasing the InSe flake thickness from a monolayer to ~30 layers, the decay time *t*_D_ of the persistent *J* gradually decreases by one order of magnitude. For bulk InSe (>100 nm), as shown in Fig. 3c, such a slow photocurrent response was not detectable (within our measurement accuracy, <200 ms). The corresponding *J*_max_ is approximately one order of magnitude less than that of the monolayer, although *C*_surf_ is dominated by *C* and is expected to be similar for both monolayer and thick InSe flakes.

The decay of the photocurrent can be described by an exponential function:

2 $$J(t) = J_0 + A \ \exp ( - kt)$$

where *J*_0_ and *A* are constants. *k* is a time constant and describes the rate of current decay. The best fit of our results using Eq. (2) demonstrated a dramatic increase in *k* when increasing the InSe crystal thickness (as shown in Fig. 3b). This indicates that the recombination of photogenerated electron–hole pairs in monolayer InSe was strongly suppressed due to the interaction between the ions and trapped holes. Similar suppressive behavior was also observed in a previous report^21^. However, in thick InSe, the total number of photo-generated holes is expected to be greater than that of the monolayers due to its reduced transparency and smaller bandgap energy^38^. The direct-to-indirect band gap transition from bulk to monolayer InSe further facilitates the photoelectric transition for thick crystals^39^. However, Fig. 3 suggests a less efficient PEC performance for the bulk InSe anode with a fewer number of photogenerated holes reacting with OH^−^ after illumination ceased. We attribute the large *k* and smaller *J*_max_ observed in thick InSe to the bulk recombination of photo-generated electrons and holes, and thus, the surface hydroxide groups trapped holes have less effect.

## Discussion

We note that the InSe/Gr heterostructure is critical to the observed high photocurrent. To demonstrate that, we performed a series of control experiments. First, although the photothermal effect has been reported to have a profound influence on the prolonged photocurrent^40,41^, we excluded its influence on the observed slow current response using few-layer InSe (e.g., Fig. 1e, 3). As demonstrated in the Supplementary Methods ‘Photothermal effects’ and Supplementary Fig. 10, we used Raman spectroscopy and found that both monolayer and thick InSe/Gr heterostructures show a similar temperature variation trend during illumination, regardless of their different current decay time constant, as shown in Fig. 3b.

Second, various interactions between the substrate and 2D heterostructures, including lattice mismatch^42,43^, hybridization^44^ and band alignment^45^, have been reported. In our case, the results were acquired from InSe/Gr on a Ti substrate supported by quartz (see Fig. 1a and “Methods” section for sample schematic). To exclude the influence of the substrate on the measured PEC performance, we investigated the InSe/Gr heterostructure on various substrates, including Ti, Cu, Au, and graphite, with distinct crystallinity and lattice constants, as shown in Fig. 1e and Supplementary Fig. 11. Both the photocurrent value and its evolution with time are comparable for all devices under the same experimental conditions, indicating that the substrate has a minor effect on the PEC performance.

Combined with DFT simulation, we attribute the high photocurrent density and persistent current after illumination to the interaction between the hydroxide ions and the photogenerated holes on the anode surface. According to the band alignment and partial density of states (PDOS) of InSe/Gr and MoS_2_/Gr heterojunctions (as shown in Supplementary Methods ‘DFT analysis’ and Supplementary Fig. 3), the lower energy of the InSe valence band facilitates the migration of photogenerated holes from the InSe to the graphene surface^46^.

Moreover, we also investigated the total energy difference (Δ*E*) before and after the reaction:

3 $${\mathrm{OH}}^ - + {\mathrm{h}}^ + \to {\mathrm{OH}}^ \ast$$

which is usually considered to be the initial step of the OER reaction. The concentration of bound hydroxide ions at the InSe/Gr surface can be estimated as *C*_surf_^InSe^ ≈ *C* exp(−Δ*E*^InSe^/*k*_B_*T*), where *k*_B_ is the Boltzmann constant. An analogous expression applies to the MoS_2_/Gr surface as well. Since *J*_max_ is proportional to *C*_surf_, it is not unreasonable to assume that *J*_max_^InSe^/*J*_max_^MoS2^ = *C*_surf_^InSe^/*C*_surf_^MoS2^. Using our results in Fig. 1e and the above expressions, we find the absolute value of Δ*E*^MoS2 ^− Δ*E*^InSe^ ≈ 0.1 eV. The lower Δ*E* for InSe/Gr compared to that of MoS_2_/Gr is supported by our DFT calculations that found Δ*E*^MoS2 ^− Δ*E*^InSe^ ≈ 0.117 eV (Supplementary Methods ‘DFT analysis’ and Supplementary Table 4). The deviation between the experiments and theory is perhaps not a surprise due to the complexity of the hydration shells of the ions in solution compare to that of ions in vacuum, which is assumed in simulations. These results indicate that the reaction of hydroxide ions with holes has a lower activation energy barrier on the surface of InSe/Gr, resulting in ion accumulation and, thus, a greater current density. For the same reason, the holes trapped by hydroxide ions on the InSe/Gr electrode surface suppress the surface electron-hole recombination, leading to the persistent current after illumination.

In conclusion, we provide insight into the strong coupling between surface adsorbed ions and photo-generated holes, which is critical for achieving highly efficient PEC water splitting. Our results demonstrate a versatile method of stabilizing chemically sensitive electrode materials and emphasize the importance of choosing specific 2D electrodes to tune ion kinetics near the electrode surface. Due to the large family of novel 2D materials and the compatibility of van der Waals heterostructures based on them^15^, these atomically thin materials with various surface properties can be used as a model system to study the mechanism of surface processes/reactions or can be integrated with conventional 3D electrodes to modify the electrode’s surface and achieve better electrochemical performance.

## Methods

### Photoanode fabrication

To avoid the degradation of the ultrathin InSe during the following measurements, exfoliation and encapsulation of the InSe photoanodes were carried out in an inert atmosphere provided by a glovebox (H_2_O and O_2_ less than 0.1 ppm). Schematic diagrams of the InSe/Gr anode fabrication process are shown in Supplementary Fig. 12. First, InSe flakes were mechanically exfoliated onto a thin polypropylene carbonate (PPC) film. Then, a monolayer InSe crystal was identified using an optical microscope and was lifted with monolayer graphene (Gr) attached to polymethyl methacrylate (PMMA) membranes using a dry peel transfer technique^21,25^. The resulting Gr–InSe stack was transferred onto a 30-nm Ti electrode with a quartz substrate prepatterned using photolithography and e-beam evaporation. The obtained heterostructures were heated at 70 °C for 1 h and then the PMMA was dissolved in acetone and isopropanol. Since the single top graphene layer is impermeable to all gas and liquid molecules^47^, such encapsulation can protect the ultrathin InSe during the following PEC measurements and prevent surface contamination. Finally, the sample was removed from the glovebox to verify the InSe thickness using atomic force microscope. Similar fabrication procedures were used for the MoS_2_/Gr anodes.

### PEC measurements

All the measurements were carried out on an electrochemical workstation (CHI 750E, Chenhua, Shanghai) using a custom-made, three-compartment photoelectrochemical cell with a Hg/HgO reference electrode and a Pt mesh counter electrode, and the electrolyte was an NaOH solution. The measured potentials were converted to the RHE scale using the equation *E*_RHE_ = *E*_Hg/HgO_ + 0.059 × pH + 0.098. Light illumination was applied at AM 1.5 G (100 mW cm^−2^) using a class AAA solar simulator (XES-40S3-TT, San-Ei Electric, Japan). Linear sweep voltammetry curves were performed at scan rate of 5 mV s^−1^. Electrochemical impedance spectroscopy (EIS) was measured by using a sinusoidal signal with amplitude of 5 mV over a frequency range of 0.1–10 kHz, and the scanning potential was 1.23 V vs. RHE.

## Supplementary information

Supplementary Information

Peer Review

## Data Availability

The data that support the findings of this study are available from the corresponding author upon reasonable request.
