# Supplementary material for: Atomically thin photoanode of InSe/graphene heterostructure
Source: Nat Commun. 2021 Jan 4;12:91. doi: 10.1038/s41467-020-20341-7 (PMC7782821; doi:10.1038/s41467-020-20341-7)
Supplement: Supplementary file 1 — Supplementary Information [file 41467_2020_20341_MOESM1_ESM.pdf]

Supporting Information

Atomically thin photoanode of InSe/graphene heterostructure

Haihong Zheng[1,5], Yizhen Lu[1,5], Kaihang Ye[2,5], Jinyuan Hu[1], Shuai Liu[1], Jiawei Yan[1], Yu Ye[3], Yuxi Guo[2], Zhan Lin[2], Jun Cheng[1,*], Yang Cao[1,4,*]

[1]State Key Laboratory of Physical Chemistry of Solid Surfaces, Collaborative Innovation Center of Chemistry for Energy Materials (*i*ChEM), College of Chemistry and Chemical Engineering, Xiamen University, Xiamen 361005, P. R. China.

[2]Guangzhou Key Laboratory of Clean Transportation Energy Chemistry, School of Chemical Engineering and Light Industry, Guangdong University of Technology, Guangzhou 510006, P. R. China.

[3]State Key Laboratory for Mesoscopic Physics, School of Physics, Peking University, Beijing 100871, P. R. China.

[4]Pen-Tung Sah Institute of Micro-Nano Science and Technology, Xiamen University, Xiamen 361005, P. R. China.

*Corresponding authors. Email: chengjun@xmu.edu.cn, yangcao@xmu.edu.cn

[5]These authors contributed equally to this work.

Supplementary Methods

DFT analysis

Computational setup: Density functional theory (DFT) calculations were carried out using the freely available program package CP2K/Quickstep[1]. For InSe, the 5s, 5p electrons of the In atom and the 4s, 4p electrons of the Se atom were treated as valence electrons. For MoS_2, the 4d, 5s electrons of the Mo atom and the 3s, 3p electrons of the S atom were treated as valence electrons. In addition, the 2s, 2p electrons of the C atom and the O atom were treated as valence electrons. The basis sets for the valence electrons consist of short-ranged (less diffuse) double-ζ basis function with one set of polarization functions (DZVP)[2]. The plane wave cutoff energy for the electron density is 400 Ry. The core electrons were represented by

analytic Goedecker-Teter-Hutter (GTH) pseudopotentials[3]. All the simulations only used the Γ point of the supercell for expansion of the orbitals considering the large size of the cell. The gradient-corrected Perdew-Burke-Ernzerhof (GGA-PBE) functional[4] was used in electronic structure calculations. We included van der Waals interaction through the using of Grimme's dispersion correction (DFT-D3)[5].

Model setup: In order to study the InSe/graphene (InSe/Gr) heterostructure, we constructed a commensurate cell by taking 15×15×1 graphene (450 atoms) and 9×9×1 InSe (324 atoms) supercell. The lattice constant mismatch is 1.06%. At the same time, 12×12×1 graphene monolayer (288 atoms) is matched to 9×9×1 MoS_2 monolayer (243 atoms), which includes the 3.4% lattice mismatch. In fact, such a negligible effect of the small lattice mismatch on the electronic structure has been well demonstrated and reported in the calculations for the similar graphene-based hybrid systems[6,7]. The slab was separated with its images by a vacuum region of 25 Å to avoid the interaction between nearest slabs. The periodic boundary conditions (PBC) were applied for the XY directions with the periodicity in the Z direction removed to eliminate the possible dipole interactions between the slabs with its images.

We first optimized the unit cell parameters based on the bulk structure, in which all atoms are relaxed. Supplementary Table 1 lists the primary cell parameters of the three structures involved in the calculation. The optimized planar lattice constant of monolayer InSe is 4.06 Å from the PBE functional, which agrees with previous experimental[8] and theoretical results[9].

Supplementary Table 1. InSe, Graphene and MoS_2 lattice constant.

System	γ-InSe	Graphene	2H-MoS_2
Structure	rhombohedral	hexagonal	hexagonal
Semiconductor	n-type	NO	n-type
Lattice constant	a= b= 4.00 Å,<br>c= 25.32 Å,<br>α= β= 90°, γ= 120°	a= b= 2.464 Å,<br>c= 6.711 Å,<br>α= β= 90°, γ= 120°	a= b= 3.161 Å,<br>c= 12.295 Å,<br>α= β= 90°, γ= 120°

In order to build a stable system and make the simulation structure consistent with the experimental structure. Firstly, we calculated the exfoliation energy of monolayer InSe and compared it with the literature results. The n-layer InSe exfoliation energy per unit area $E_{exf}(n)$ can be calculated by Supplementary Equation 1[10]:

$$E_{exf}(n) = \frac{E_{iso}(n) - \frac{n}{m}E_{bulk}}{A} \qquad \text{(Supplementary Equation 1)}$$

Where $E_{iso}(n)$ is the energy of the unit cell of an isolated n-layer slab in vacuum, E_{bulk} is the energy of a bulk material with m layers, and A is the in-plane area of the bulk unit cell. Using the corresponding values as shown in Supplementary Table 2, the exfoliation energy of InSe we calculated is 14.5 meV A^{-2}, which is agree with the reported binding energy of 14.9 meV A^{-2} for monolayer InSe[11] and proved InSe is easy to exfoliate.

Supplementary Table 2. Exfoliation energy of monolayer InSe.

System	E_{iso} (eV)	E_{bulk} (eV)	A (Å^2)	n	m	E_{exf} (meV A^{-2})
InSe	-14.797	-45.109	16.456	1	3	14.5

Secondly, one of the reasons we use graphene is that the surface of graphene easily adsorbs hydroxide ions. We calculated the adsorption energy (E_{ad}) of hydroxide ions on the surface of InSe/Gr by the following formula:

$$E_{ad} = E(OH_a) - E(OH_f) \qquad \text{(Supplementary Equation 2)}$$

Where the indices a and f stand for adsorbed hydroxide ions and desorbed hydroxide ions, respectively. The adsorption energy of hydroxide ions on InSe/Gr is -0.74 eV.

Thirdly, we extract the Interlayer distance ($d_{InSe/Gr}$) between monolayer InSe and graphene from the lowest point of the distance dependence of binding energy curves as shown in Supplementary Fig. 2. The lowest point of energy is the most stable state, which value can be calculated according to the following formula:

$$E_b = E_{InSe/Gr} - (E_{InSe} + E_{Gr}) \qquad \text{(Supplementary Equation 3)}$$

Where E_b is the binding energy of monolayer InSe and graphene. $E_{InSe/Gr}$, E_{InSe} and E_{Gr} represents the energy of InSe/Gr heterostructure, InSe and graphene, respectively. We used the same method to obtain interlayer distance of monolayer MoS_2 and graphene. The values are 3.44 Å for $d_{InSe/Gr}$ and 3.36 Å for $d_{MoS2/Gr}$, respectively, in good agreement with the values of 3.3-3.4 Å reported from both theoretical calculation and cross-section characterization of typical graphene and transition metal dichalcogenide (TMDC) interlayer distance[12-14].

Band alignment of InSe/Gr and MoS_2/Gr: In order to obtain the energy band alignment of the heterostructure, we calculated the band structure based on the constructed heterostructures, as shown in Supplementary Table 3 and Supplementary Fig. 3. We consider the band alignment taking the electron affinities and work functions into account, but neglecting the possible presence of defects and/or impurities at the interface. The work function of the heterostructure is obtained by the following formula:

$$\phi = E_{vac} - E_{Fermi} \qquad \text{(Supplementary Equation 4)}$$

where E_{vac} represents the energy of the vacuum level, E_{Fermi} represents the energy of the Fermi level and ϕ represents the work function. The band structure data of InSe/Gr and MoS_2/Gr are listed in Supplementary Table 3.

Supplementary Table 3. Band structures of InSe/Gr and MoS_2/Gr.

System	E_{vac} (eV)	E_{Fermi} (eV)	ϕ (eV)
InSe/Gr heterostructure	0.06	-4.57	4.63
MoS_2/Gr heterostructure	0.08	-4.60	4.68

To compare the difference in hole transport between two heterostructures, we use the partial density of states (PDOS) to analyze the relative band alignment of InSe/Gr and MoS_2/Gr. When referring the energy levels to the Fermi levels of graphene, the energy position of the valence band maximum (VBM) of MoS_2 is higher than that of InSe ($\Delta'_{VB} = E^{MoS_2}_{VBM} - E^{InSe}_{VBM}$ = 0.29 eV) as shown in Supplementary Fig. 3. Thus, the lower energy of InSe valence band facilitates the migration of photo-generated holes from InSe to the graphene surface[15]. We suspect that the difference of the VBM energy position will affect the total energy change before and after the hydroxide ions and holes reaction.

Total energy change before and after the reaction of hydroxide ions and holes: In order to quantitatively study the above suspension, we calculated the energy change (ΔE) of the reaction between hydroxide ions and holes according to the following formula[16]:

$$OH^- + h^+ \rightarrow OH^* \qquad \text{(Supplementary Equation 5)}$$

Where the reactants of hydroxide ions (OH^-) come from the electrolyte, and the holes (h^+) come from semiconducting InSe or MoS_2 and then transfer to the surface of graphene. The product OH* stand for the adsorbed hydroxide ions on the surface of InSe/Gr or MoS_2/Gr. For the reactants, we first added a OH^- to

the system (the distance of OH^- from the graphene surface is approximately 2 Å), the initial structure of hydroxide ions near the InSe/Gr and MoS_2/Gr anode as shown in Supplementary Fig. 4. Then the atoms were relaxed to optimize. Based on this structure, a h^+ was introduced into the system and optimized again to obtain the final stable structure. For the products, we introduced a OH* and optimized it to obtain the final stable structure as shown in Fig. 1c.

The ΔE before and after the reaction is the energy difference of the products (OH*) and the reactants (OH^- and h^+). The ΔE was calculated by Supplementary Equation 6 and the results are shown in Supplementary Table 4. The lower value of ΔE^{InSe} compare to that of ΔE^{MoS2} indicates the reaction on InSe/Gr is easier to conduct.

$$\Delta E = E_{products} - E_{reactants} \qquad \text{(Supplementary Equation 6)}$$

Supplementary Table 4. Total energy change (ΔE) on InSe/Gr and MoS_2/Gr before and after the reaction. ΔE^{MoS2}, ΔE^{InSe} and ΔE^{MoS2} - ΔE^{InSe} represent the ΔE on MoS_2/Gr anode, InSe/Gr anode and their difference, respectively.

System	ΔE (eV)	ΔE^{MoS2} - ΔE^{InSe} (eV)
InSe/Gr heterostructure	0.462	0.117
MoS_2/Gr heterostructure	0.579	

EIS

The analysis of electrochemical impedance spectroscopy (EIS) is based on the equivalent circuits presented in the inset of Fig. 2b, where the symbol R_s, represents the solution resistance, R and R_{ct} represent the resistances at the interface between Ti/photoanode and the photoanode/electrolyte, respectively. C_{dl} is the electrical double-layer capacitance of photoanode/electrolyte interface and CPE (Constant Phase Element) is the constant phase element standing for Ti/photoanode. Dimensionless constant n in the CPE defines the interfacial structures varying from resistor-like (n~0) to capacitor-like (n~1). From the EIS data of InSe/Gr, we found two clearly semi-circles, the diameter of which can represent the well-defined photoanode/electrolyte electric double layer (EDL) interfacial capacitance (C_{dl}) and the capacitor-like structure capacitance by Ti/photoanode (CPE). To make it simple, we use the same equivalent electrical circuit with the same meaning to describe the interfacial process. From the fitting

results, n (MoS_2/Gr) is almost 0, which means only the EDL significantly affects the interfacial capacitance (C_{dl}).

Supplementary Table 5. Area-normalized capacitances of the EDL.

C_A	Light off (F cm^{-2})	Light on (F cm^{-2})
InSe/Gr heterostructure	0.06	0.14
MoS_2/Gr heterostructure	0.0018	0.0017

From Fig. 2b, we can also estimate the value of interface capacitance (C_A) of the electrical double layer (EDL) from the diameter of the semi-circles and the results shown in Supplementary Table 5. According to the equation: $C_A = \varepsilon_0\varepsilon_r/d_1$, where ε_0 is the dielectric constant in vacuum, ε_r is the relative dielectric constant of the electrolyte, d_1 is the charge separation distance (m). ε_0 and ε_r are constant in our system, so the bigger capacitance may correspond to a shorter d_1. In previous reports, it has been seen that attractive interactions between ions and graphene that narrow the double layer, decreasing the bias voltage necessary to drive charge separation and enhancing the EDL capacitance[17]. In our system, under illumination, the capacitance at the interface of InSe/Gr and solution is ~ 2.3 times higher than that when illumination off shown in Supplementary Table 5. It may because the amount of OH^- adsorbed on graphene surface increased due to more photo-generated holes, thus the thickness of the electrical double layer decrease. While for MoS_2/Gr, C_A did not change much when illumination on or off, and the value is almost two orders of magnitude smaller than the one of InSe/Gr. This indicates that it is hard for the surface of MoS_2/Gr to adsorb OH^-.

Photothermal effects

Note photothermal effects have been reported to have profound influence to photocurrent response, in order to study their influence, we calculated and compared the temperature of the monolayer and bulk InSe/Gr heterostructures by detecting Raman spectra of the top graphene, since the G peak frequency of graphene in sensitive to the temperature[18,19].

Raman spectra were recorded on a laser micro-Raman spectrometer (Renishaw inVia). All spectra were excited with visible (532 nm) laser light and a 50x long working distance air objective (laser spot size ~ 2 µm). A cold-hot cell operated using a liquid nitrogen source was used to control the temperature in the

Raman experiments with 10 °C min^{-1} of heating/freezing rates. The accuracy and stability of this system is 0.1 °C. Prior to measurements, samples should be balanced for at least 30 min after each temperature of interest was reached.

At first, the dependence of the G peak frequency of graphene on the temperature (Supplementary Fig. 10b) was plotted with linear fit as the work curve (Supplementary Fig. 10a). To detect the real temperature of the InSe/Gr heterostructure during the illumination on or off, the samples were illuminating at room temperature and the Raman shifts were recorded at fixed time intervals. The illumination source was a 300 W Xe arc lamp (Newport, 6255) with a power density of about 100 mW cm^{-2} and an AM 1.5 G filter was used to obtain simulated sunlight, consistent with our PEC measurements. These Raman curves were then compared with the work curve in Supplementary Fig. 10a to calculate the temperature on the heterostructure surface.

The dependence of the temperature on the illumination time is shown in Supplementary Fig. 10c – 10f. The results indicate that the temperature of monolayer InSe/Gr surface increased from 24 to almost 90 °C when the surface was illuminated from 0 to 20 min, and then kept constant until the light off. And the temperature slowly dropped to room temperature, taking about 40 min after light off. We used the same method to calculate the temperature for the bulk InSe/Gr. The curve shows a similar temperature variation trend. The results suggest that photothermal effect is not the dominating factor for the different behaviors with various InSe thicknesses (Fig. 3) and for the slow current response in monolayer InSe/Gr photoanodes.

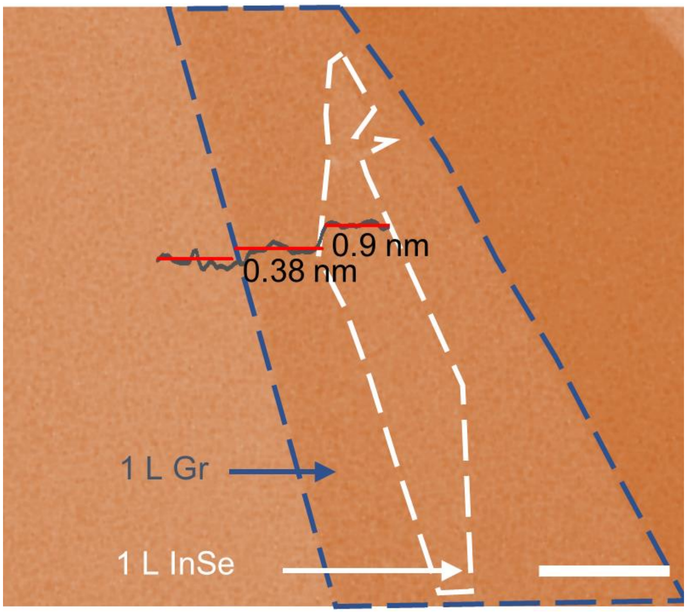

Supplementary Figure 1. Atomic force microscope (AFM) characterization and optical image of a representative monolayer InSe/Gr heterostructure. The blue and white contours indicate the position of monolayer graphene and monolayer InSe, respectively. The scale bar is 20 μm.

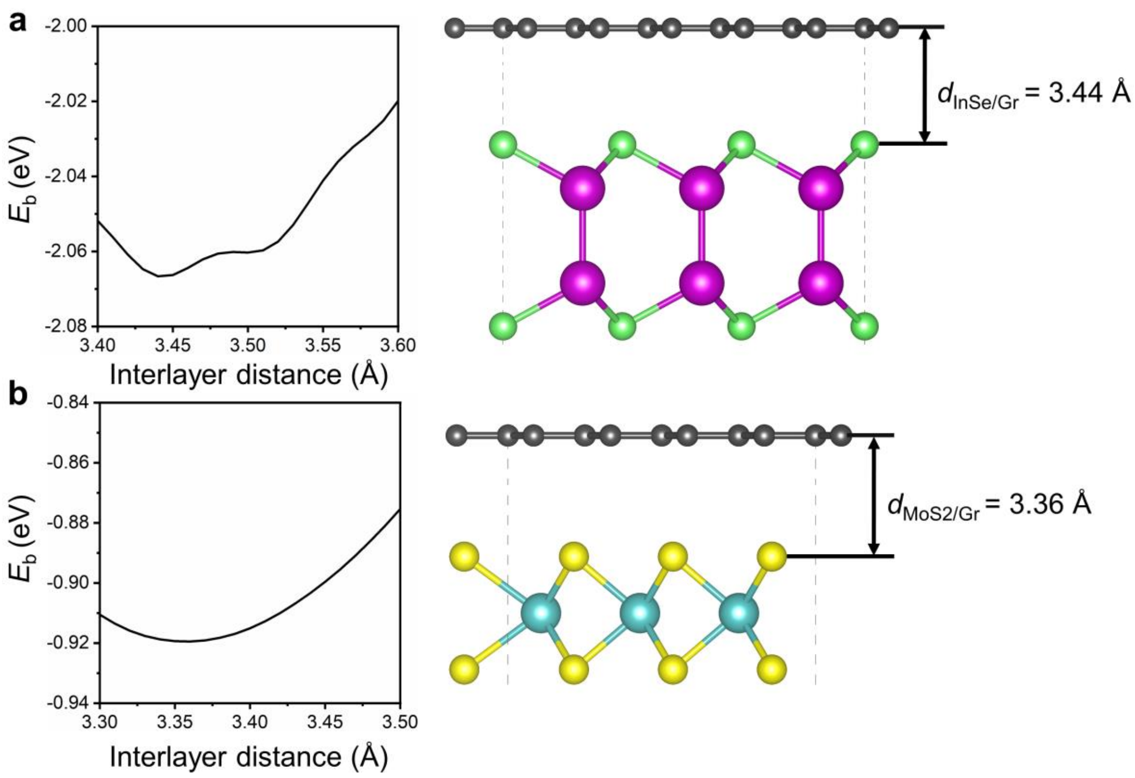

Supplementary Figure 2. Interlayer distance of InSe/Gr and MoS_2/Gr heterostructure. Binding energy of InSe/Gr and MoS_2/Gr heterostructure as a function of the interlayer spacing between graphene and the topmost atom of InSe or MoS_2: **a** For InSe/Gr system; **b** For MoS_2/Gr system. All the atomic positions of InSe/Gr systems were fully relaxed during structural optimization.

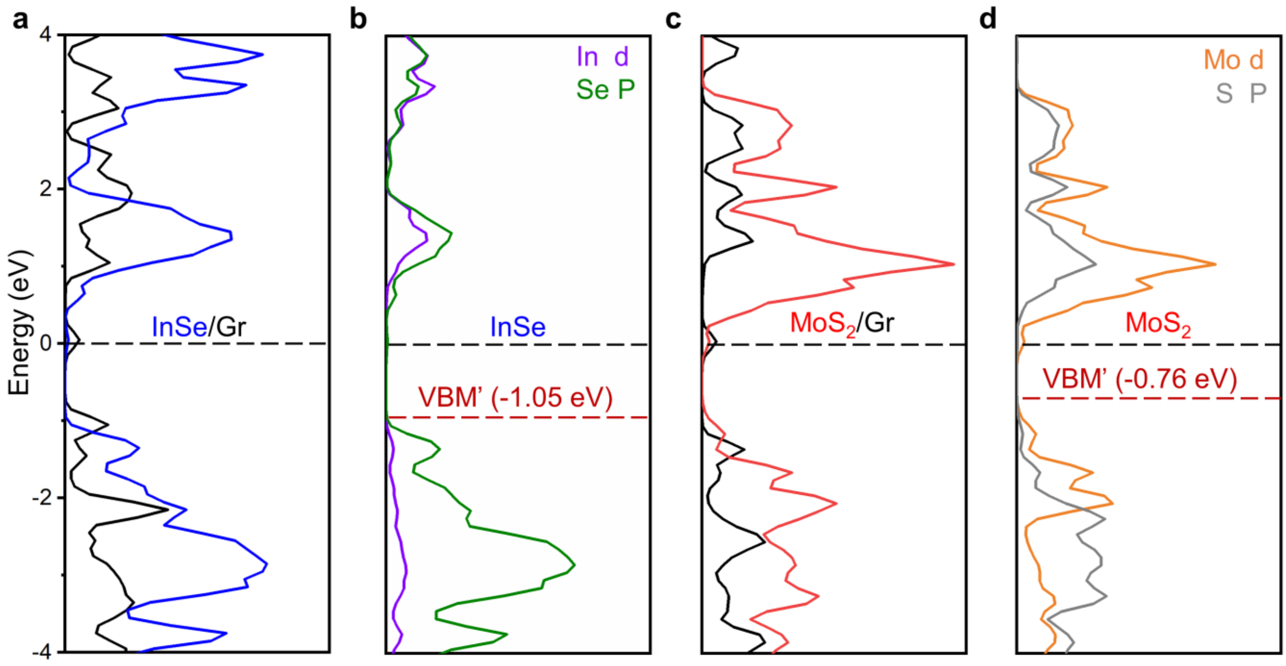

Supplementary Figure 3. Partial density of states (PDOS) of InSe/Gr and MoS_2/Gr heterostructures. **a** PDOS of InSe/Gr. Black, blue lines represent graphene and InSe, respectively. **b** PDOS of d orbitals of In atoms (purple) and p orbitals of Se atoms (green). **c** PDOS of MoS_2/Gr. Black, red lines represent graphene and MoS_2, respectively. **d** PDOS of d orbitals of Mo atoms (orange) and p orbitals of S atoms (gray). All the energy levels are referenced to the fermi energy of graphene.

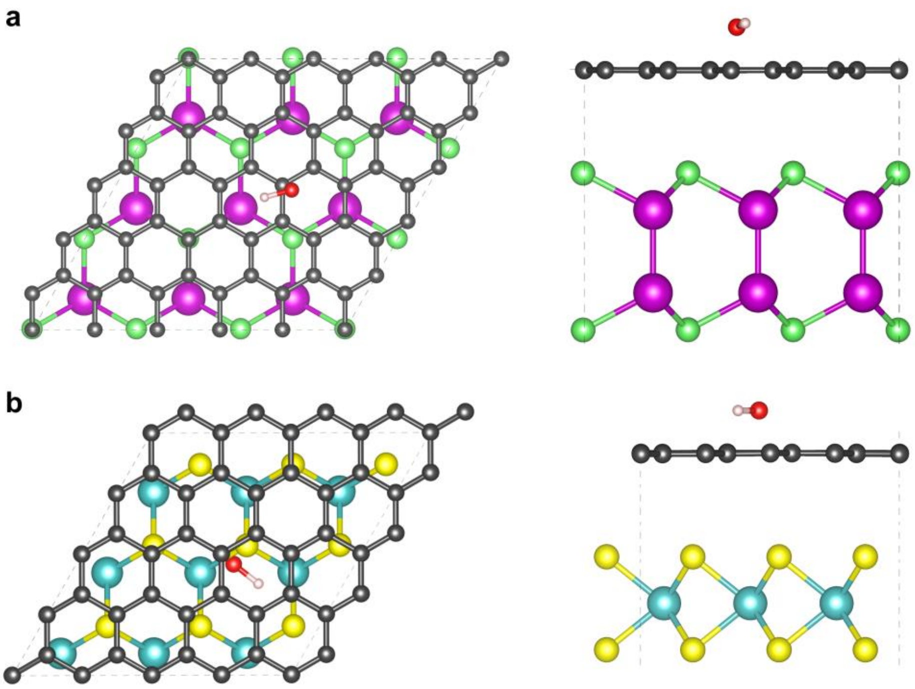

Supplementary Figure 4. Initial structure of hydroxide ions near the InSe/Gr and MoS_2/Gr anode. **a** For InSe/Gr system; **b** For MoS_2/Gr system. Left, top view. Right, side view. Red, white, gray, purple, green, cyan, yellow balls represent oxygen, hydrogen, carbon, indium, selenium, molybdenum and sulfur atoms, respectively.

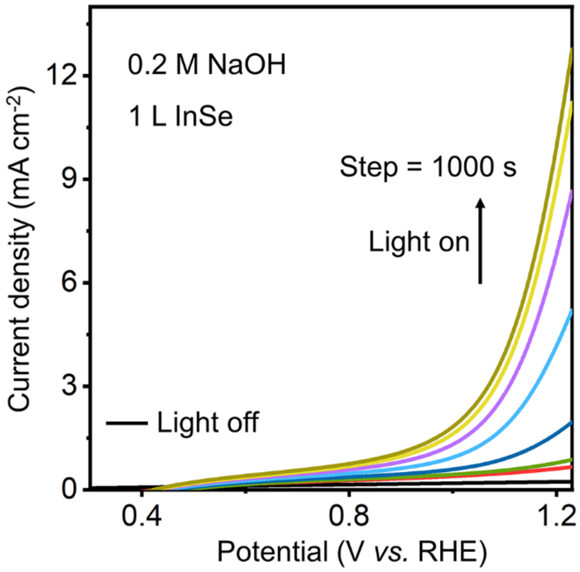

Supplementary Figure 5. LSV curves of InSe/Gr anode. The original linear-scale curves of the one in Fig. 1d: Linear sweep voltammetry of a representative monolayer InSe/Gr device measured at different illumination time.

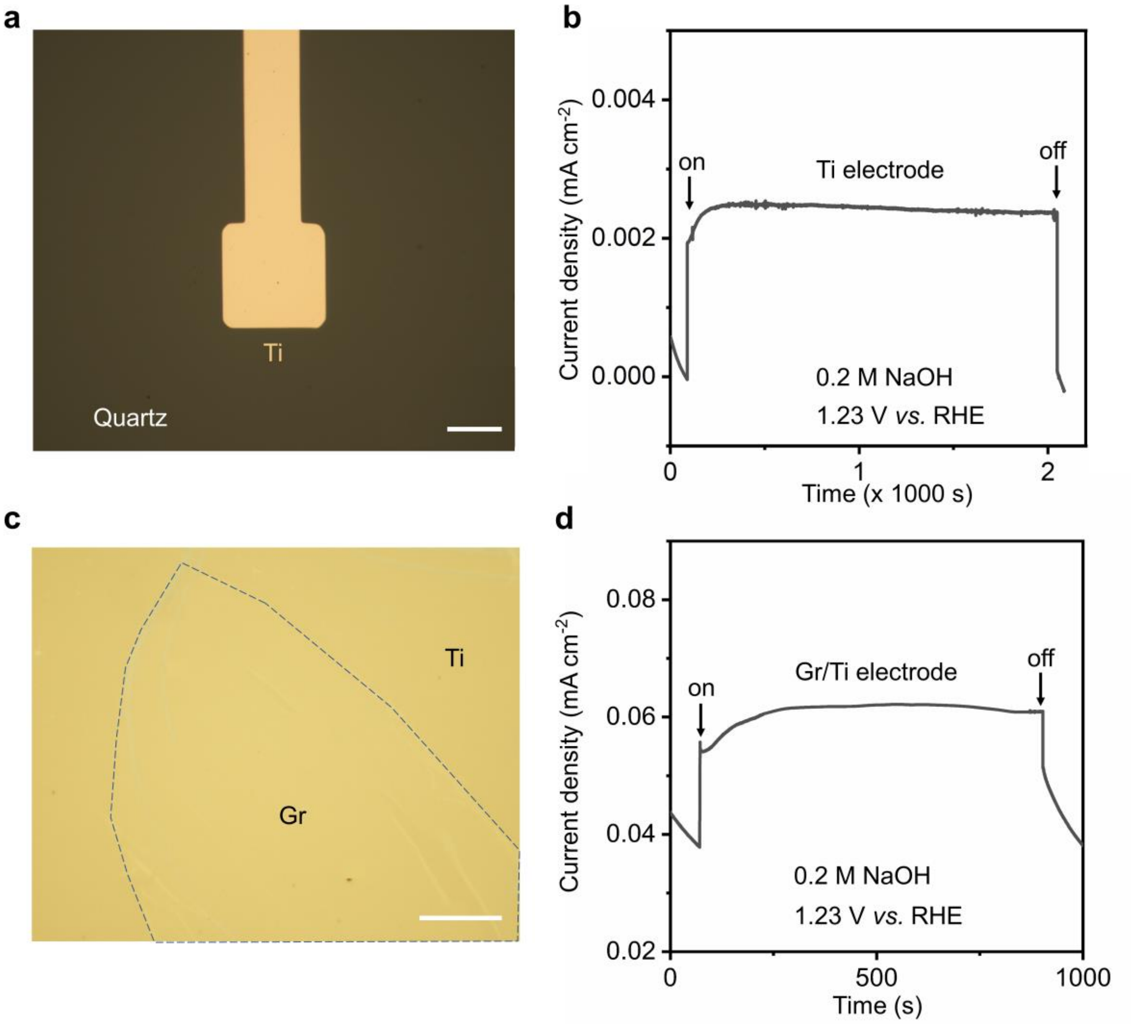

Supplementary Figure 6. Optical images and PEC measurements of Ti electrode and graphene/Ti electrode. **a** Optical image of Ti electrode, the scale bar is 50 μm. **b** Photo current density of Ti electrode as a function of time when light on or off. J_{max} is about 0.0025 mA cm^{-2}. **c** Optical image of graphene/Ti electrode, the blue contours indicate the area of monolayer graphene. The scale bar is 20 μm. **d** Photo current density of graphene/Ti electrode as a function of time when light on or off. J_{max} is about 0.024 mA cm^{-2}.

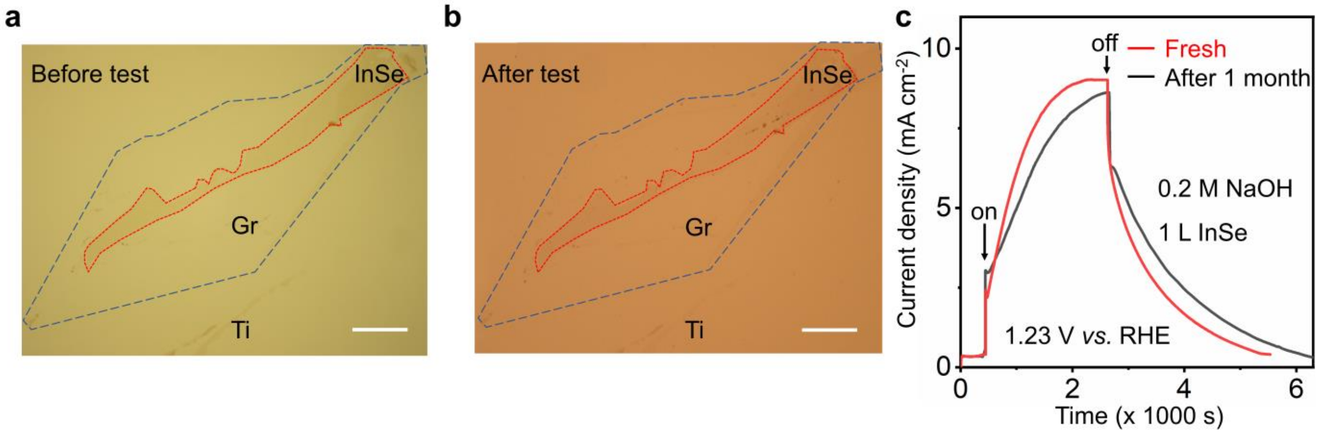

Supplementary Figure 7. Stability of InSe/Gr anode. Optical image of monolayer InSe/Gr heterostructures: **a** Before test; **b** After test. The blue and red contours indicate the position of monolayer graphene and monolayer InSe, respectively. The scale bars are 20 μm. After test, there is no discernable damage on both InSe and graphene. **c** Photo current density of freshly assembled and one-month atmosphere-aged monolayer InSe/Gr anode as a function of time when light on or off. The fresh anode shows the J_{max} is about 8.8 mA cm^{-2}. After 1 month leaving in air, the anode shows the J_{max} is about 8.4 mA cm^{-2}, little variation (< 5%) of their original value. Note the good sticking property between Ti and quartz also promotes the good stability of the photoanode[20].

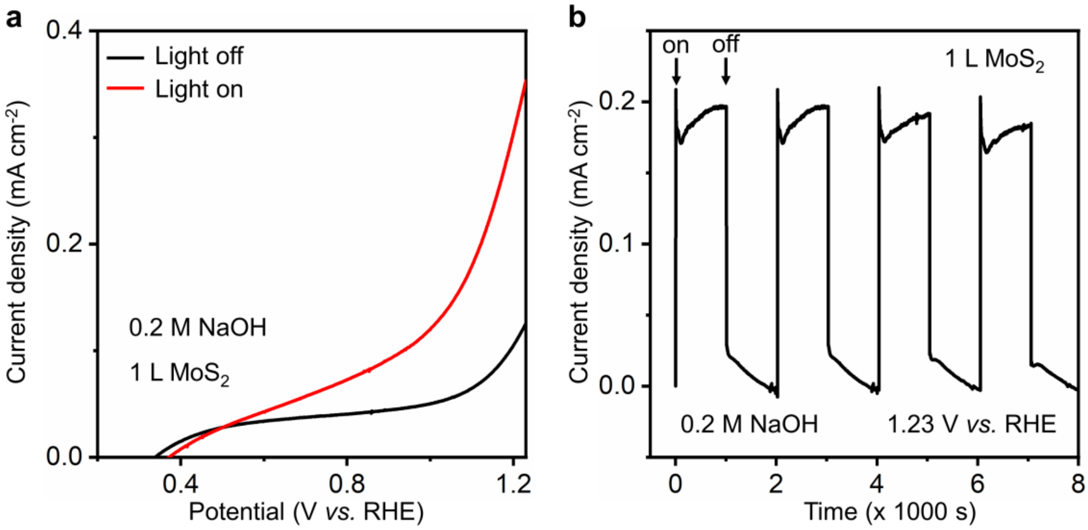

Supplementary Figure 8. MoS_2/Gr anode for PEC measurements. **a** Linear sweep voltammetry of a representative monolayer MoS_2/Gr device measured when light on or off. **b** Photo current density of PEC cell as a function of time when light on or off. Under light illumination, the linear sweep voltammetry (LSV) shows a hydroxide ion oxidation onset potential of ~ 380 mV. The measured photo current density *J* is ~ 0.2 mA cm^{-2}.

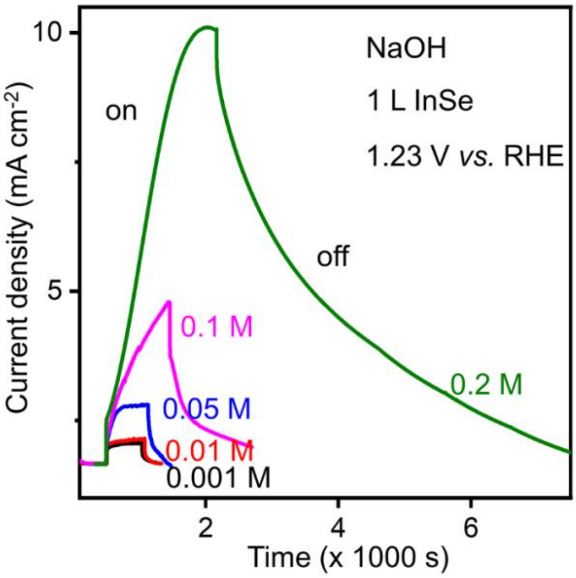

Supplementary Figure 9. Current density of monolayer InSe/Gr anode as a function of NaOH concentration.

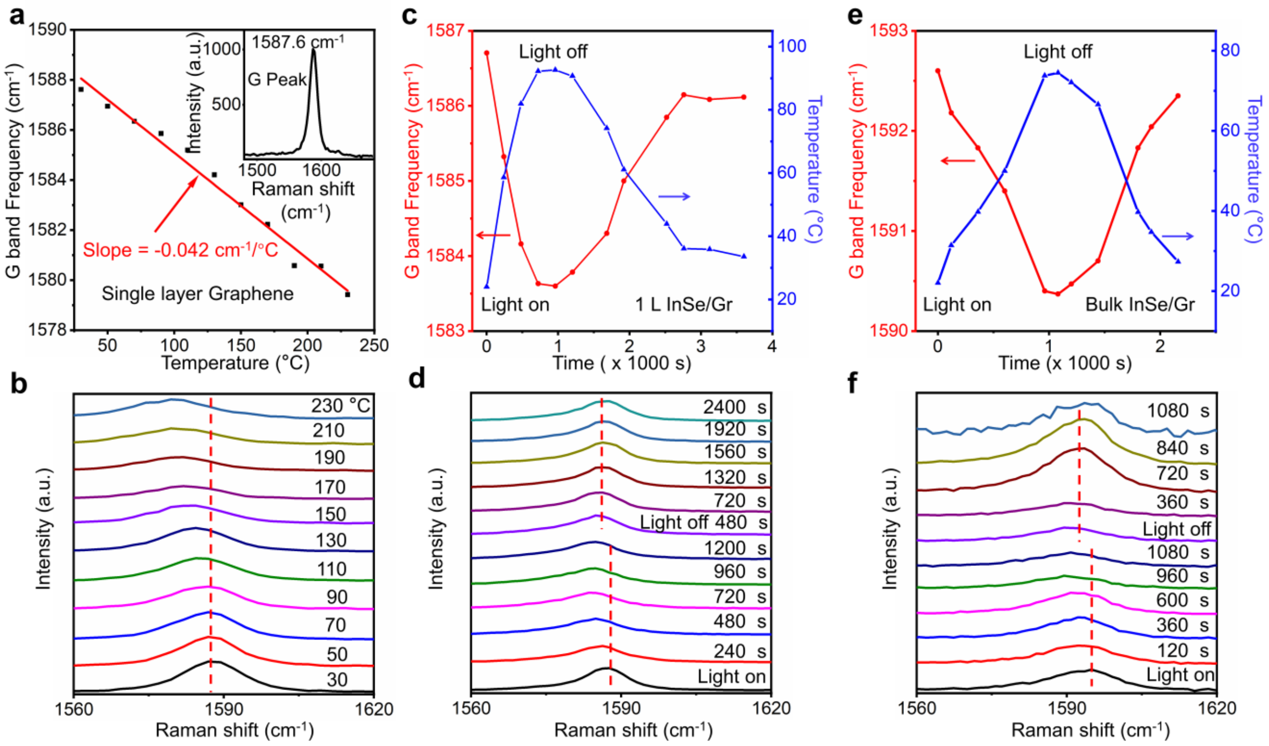

Supplementary Figure 10. Photothermal effect for 1L InSe/Gr and bulk InSe/Gr. **a** Temperature dependence of the G peak frequency for the single layer graphene. The inset shows the shape of G peak. The measured data were used to extract the temperature coefficient for G peak. **b** Raman spectra of the single layer graphene with different temperature. **c, e** The dependence of G peak frequency (red) and temperature (blue) on the illumination time for graphene on single layer InSe (**c**) and bulk InSe (**e**), respectively. **d, f** Raman spectra of graphene on single layer InSe (**d**) and bulk InSe (**f**) with various illumination time, respectively.

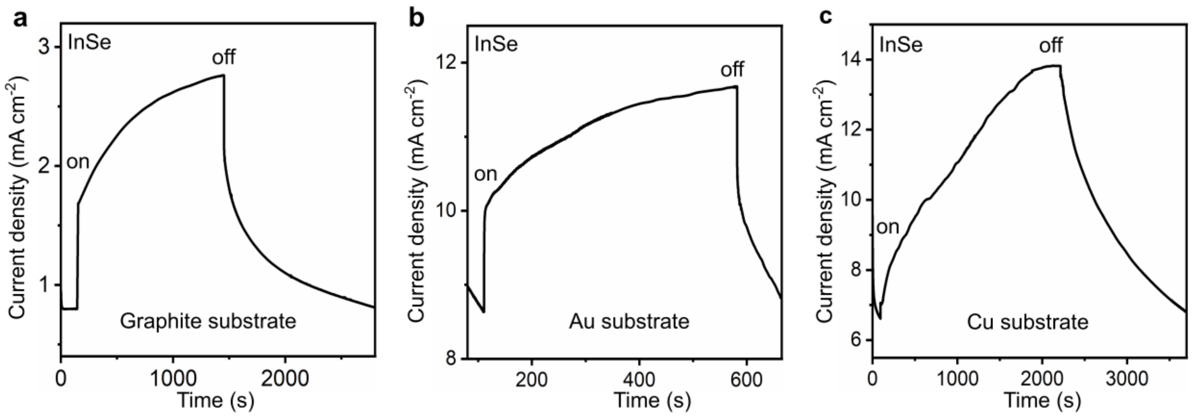

Supplementary Figure 11. Photocurrent density of InSe/Gr photoanode on different substrates as a function of time when light on or off. **a** Single crystalline graphite substrate, **b** Polycrystalline Au substrate, **c** Polycrystalline Cu substrate. *J-t* curves are measured at potential 1.23 V *vs.* RHE and in 0.2 M NaOH solutions.

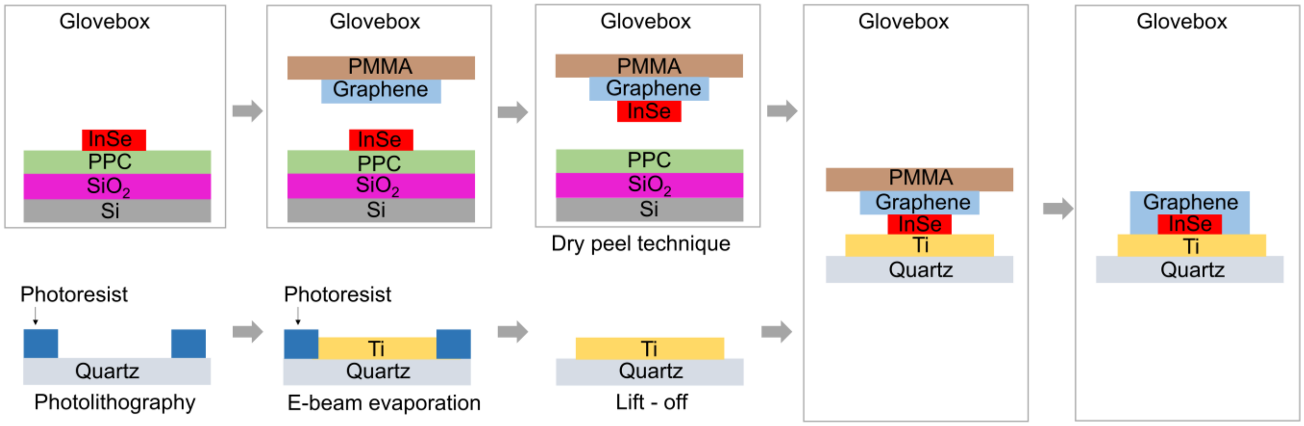

Supplementary Figure 12. Schematic diagram of InSe/Gr anode fabrication process.

Supplementary References

1 VandeVondele, J. *et al.* QUICKSTEP: fast and accurate density functional calculations using a mixed Gaussian and plane waves approach. *Comput. Phys. Commun.* **167,** 103-128 (2005).

2 VandeVondele, J., Hutter, J. Gaussian basis sets for accurate calculations on molecular systems in gas and condensed phases. *J. Chem. Phys.* **127,** 114105:1-9 (2007).

3 Hartwigsen, C., Goedecker, S., Hutter, J. Relativistic separable dual-space Gaussian pseudopotentials from H to Rn. *Phys. Rev. B*, **58,** 3641–3662 (1998).

4 Perdew, J. P., Burke, K., Ernzerhof, M. Generalized Gradient Approximation Made Simple. *Phys. Rev. Lett.* ***77*****,** 3865–3868 (1996).

5 Grimme, S., Antony, J., Ehrlich, S., Krieg, H. A consistent and accurate ab initio parametrization of density functional dispersion correction (DFT-D) for the 94 elements H-Pu. *J. Chem. Phys.* **132**, 154104:1-18 (2010).

6 Liu, Y., Huang, Y. & Duan, X. Van der Waals integration before and beyond two-dimensional materials. *Nature* **567,** 323-333 (2019).

7 Kistanov, A. A., Cai, Y., Zhou, K., Dmitriev, S. V., Zhang, Y.-W. Effects of graphene/BN encapsulation, surface functionalization and molecular adsorption on the electronic properties of layered InSe: a first-principles study. *Phys. Chem. Chem. Phys.* **20**, 12939-12947 (2018).

8 Lei, S., *et al.* Evolution of the Electronic Band Structure and Efficient Photo-Detection in Atomic Layers of InSe. *ACS Nano* **8,** 1263–1272 (2014).

9 Shen, T., Ren, J. C., Liu, X., Li, S., Liu, W. Van der Waals stacking induced transition from schottky to ohmic contacts: 2D metals on multilayer InSe. *J. Am. Chem. Soc.* **141,** 3110-3115 (2019).

10 Li, T., He, C., Zhang, W. Two-dimensional porous transition metal organic framework materials with strongly anchoring ability as lithium-sulfur cathode. *Energy Stor. Mater.* **25,** 866-875 (2020).

11 Mounet, N., *et al.* Two-dimensional materials from high-throughput computational exfoliation of experimentally known compounds. *Nat. Nanotechnol.* **13,** 246-252 (2018).

12 Gmitra, M., Kochan, D., Hogl, P., Fabian, J. Trivial and inverted dirac bands and the emergence of quantum spin Hall states in graphene on transition-metal dichalcogenides. *Phys. Rev. B* **93,** 155104:1-10 (2016).

13 Novoselov, K. S., Mishchenko, A., Carvalho, A., Castro Neto, A. H. 2D materials and van der Waals

heterostructures. *Science* **353,** aac9439:1-11 (2016).

14 Cui, X. *et al.* Multi-terminal transport measurements of MoS_2 using a van der Waals heterostructure device platform. *Nat. Nanotechnol.* **10,** 534–540 (2015).

15 Moniz, S. J. A., Shevlin, S. A., Martin, D. J.,Guo, Z.-X., Tang, J. W. Visible-light driven heterojunction photocatalysts for water splitting – a critical review. *Energy Environ. Sci.* **8,** 731-759 (2015).

16 Huang, Z.-F. *et al.* Design of Efficient Bifunctional Oxygen Reduction/Evolution Electrocatalyst: Recent Advances and Perspectives. *Adv. Funct. Mater.* **7,** 1700544:1-21 (2017).

17 Ji, H. *et al.* Capacitance of carbon-based electrical double-layer capacitors. *Nat. Commun.* **5,** 3317:1-7 (2014).

18 Zhan, C. *et al.* Disentangling charge carrier from photothermal effects in plasmonic metal nanostructures. *Nat. Commun.* **10,** 2671:1-8 (2019).

19 Yang, H. *et al.* Tunable wavelength enhanced photoelectrochemical cells from surface plasmon resonance. *J. Am. Chem. Soc.* **138,** 16204-16207 (2016).

20 Kim, Y.-H., Chaug, Y. S., Chou, N. J., Kim. J. Adhesion of titanium thin film to oxide substrates. *J. Vac. Sci. Technol. A* **5,** 2890-2893 (1987).
