# Supplementary material for: Atomically thin photoanode of InSe/graphene heterostructure
Source: Nat Commun. 2021 Jan 4;12:91. doi: 10.1038/s41467-020-20341-7 (PMC7782821; doi:10.1038/s41467-020-20341-7)
Supplement: Supplementary file 2 — Peer Review [file 41467_2020_20341_MOESM2_ESM.pdf]

Reviewer #1 (Remarks to the Author):

The manuscript with reference number “NCOMMS-20-24550” entitled “Ultrahigh current density on atomically thin photoanode of InSe/graphene heterostructure” presents an ultrahigh current density of atomically thin InSe crystals encapsulated with monolayer graphene to ensure high stability as photoanode for water splitting application. The manuscript utilized various materials characterization techniques as well as DFT computational study for band structure determination. A maximum current density of about 10 mA/cm2 for the photoanodes was measured with a good life time of about one month that confirms the stability of InSe crystals with graphene encapsulation. But, it needs to be further improved before publication. My comments are as follows:

1-Since the InSe layer thickness is very low, the concept of lattice mismatch is an important issue in device application and stability. What is the measured lattice mismatch between InSe monolayer and Ti substrate?

2-What are the relaxed lattice constants of the deposited monolayer InSe?

3-Band gap energy strongly depends on any semiconductor film thickness. Please discuss the band gap energy variation with the InSe layer thickness.

4- Please discuss the interaction and sticking property between Ti and Quartz substrate (two different in nature, one is metal and other is insulator as glass).

5-Please discuss the nature (direct/indirect) of InSe band gap energy with the deposited InSe layer thicknesses.

6-It is well known that there exists a strong hybridization between Ti and InSe. There is no any discussion on this concept. Please discuss this issue with appropriate updated references.

7-In Page 6, line 146, (CPE) should be introduced for the first time. It was introduced in supporting document. It should be written as” Constant Phase Element (CPE) in the main text as well”

8-In some references, the last page number of articles is missing. To be consistent with the entire manuscript, please add last page.

9-There is only one reference published in 2020, please update and add some new relevant references

Reviewer #2 (Remarks to the Author):

Authors provided an insight of strong coupling between surface adsorbed ions and photogenerated holes, which is critical for achieving highly efficient PEC water splitting. The results demonstrated a versatile method of stabilizing chemical sensitive electrodes materials and emphasized the importance of choosing specific 2D electrodes to tune ions concentration near electrode surface. The manuscript is well organized and conclusions are well supported on calculation results. I recommend the publication of the present work after revisions.

1.The author should explain the difference in scale between experiment and simulation, and how to make the results of experiment and simulation confirm each other. Specifically, how to prove that the simulation structure is consistent with the experimental structure?

2.The exfoliation energies of InSe monolayer should be mentioned in Supplementary Materials (Refer to Energy Storage Materials 25 (2020) 866–875)

3.In Fig.1c, 4. how to determine the adsorption position of hydroxide ions on the InSe/Gr heterostructure surface? Is the process of multiple hydroxide ions considered, and does the author

consider the change of free energy in the process of water splitting?
4. Why do authors choose to discuss the situation when pH = 13?

Reviewer #3 (Remarks to the Author):

The authors present in this article the deposition, analysis and application of InSe crystals protected by graphene. The photocurrent densities obtained are sound and they present useful and interesting characterisation. However, the work does not seem complete enough and in the typical format and detail of NAture Communications. For example:
- There is no paragraph introducing the paper at the end of the introduction.
- There is no discussion.
- The Experimental does not have many details, especially in the deposition of the films (could this paper be reproduced by a PhD student? Are there more crystals in the electrode contributing to the photocurrent? How is the area calculated ?).
- Some wording could be improved or corrected (wrong use of "logit", "virgin curves" better simply called "linear-scale curves" ...)
- Fig 3c would be read better in a 2D graph

Responses to the reviewers' comments:

Reply to Reviewer #1

The manuscript with reference number "NCOMMS-20-24550" entitled "Ultrahigh current density on atomically thin photoanode of InSe/graphene heterostructure" presents an ultrahigh current density of atomically thin InSe crystals encapsulated with monolayer graphene to ensure high stability as photoanode for water splitting application. The manuscript utilized various materials characterization techniques as well as DFT computational study for band structure determination. A maximum current density of about 10 mA/cm2 for the photoanodes was measured with a good life time of about one month that confirms the stability of InSe crystals with graphene encapsulation. But, it needs to be further improved before publication. My comments are as follows:

Response:

Thank you very much for your good comments.

1-Since the InSe layer thickness is very low, the concept of lattice mismatch is an important issue in device application and stability. What is the measured lattice mismatch between InSe monolayer and Ti substrate?

Response:

Thank you for pointing out the issue of lattice mismatch. Indeed, lattice mismatch occurs when bring two materials with different lattice constant into contact [*Nat. Mater* **3**, 769–773 (2004)], and it is critical for thin films, especially for 2D materials, based devices' performance.

In our case, however, it is difficult to define and evaluate the lattice mismatch on the whole sample between InSe and Ti substrates, simply because the Ti substrate is polycrystalline. The Ti substrate used to support InSe monolayers is deposited by e-beam evaporation. X-ray diffraction result (Fig.R1) shows that the film is poly-crystal, in consistent with the results reported from literature [*Nature* **567**, 323–333 (2019), *Coatings* **10**, 443:1-10 (2019)]. When InSe crystal is assembled on top of Ti film by van der Waals force, the contacted Ti surface is variously oriented.

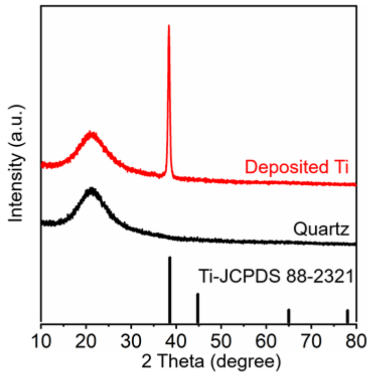

Figure R1. XRD pattern of the deposited Ti electrode (100 nm Ti on 1 mm quartz substrate), quartz substrate and standard pattern of Ti (JCPDS 88-2321).

Nevertheless, in order to investigate the influence of lattice mismatch to the heterostructures' PEC performance, we compared results from devices of InSe/Gr deposited on various substrates including single crystalline graphite, polycrystalline Au and Cu films. As shown in Fig. R2, despite the substrates have distinct lattice constant and thus different lattice mismatch with InSe, both the photocurrent value and its evolution with time are comparable for all devices under the same experimental conditions. Thus our results indicate that the lattice mismatch is a minor factor to the PEC performance.

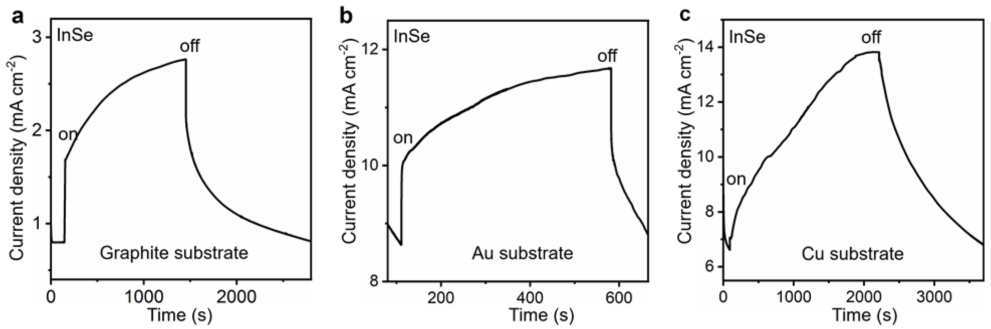

Figure R2. Photocurrent density of InSe/Gr photoanode on different substrates as a function of time when light on or off. **a** Single crystalline graphite substrate, **b** Polycrystalline Au substrate, **c** Polycrystalline Cu substrate. *J-t* curves are measured at potential 1.23 V *vs.* RHE and in 0.2 M NaOH solutions.

We have added the above discussion of lattice mismatch to the revised main text page 9, line 226 "Second, it has been reported that there are various interactions between substrate and 2D materials heterostructures including lattice mismatch[42,43], hybridization[44] and band alignment[45]. In our case, the reported results were acquired from InSe/Gr on Ti substrate supported by quartz (see Fig. 1a and

Methods for sample schematic). To exclude the influence of substrate to the measured PEC performance, we investigated InSe/Gr heterostructure on various substrates including Ti, Cu, Au and graphite with distinct crystallinity and lattice constant as shown in Fig.1e and Supplementary Fig. 11. Both the photocurrent value and its evolution with time are comparable for all devices under the same experimental conditions, indicating that the substrate has minor effect to the PEC performance.", and have added the related Figures in page 15, line 266 in Supplementary Materials.

2-What are the relaxed lattice constants of the deposited monolayer InSe?

Response:

The primitive lattice constant for bulk-InSe we used in DFT simulation is: a= b= 4.00 Å, c= 25.32Å, α= β= 90°, γ= 120°, as shown in Fig. R3, referring to the literature *Acta Crystallogr. B* **31**, 1252-1254 (1975). Based on this, for monolayer InSe, the optimized planar lattice constant of both a and b axes is 4.06 Å, which calculated by Perdew-Burke-Ernzerhof (PBE) functional [*Phys. Rev. Lett.* **77**, 3865–3868 (1996)]. The value agrees with previous experimental reports [*J. Cryst. Growth* **57**, 482–486 (1982), *ACS Nano* **8**, 1263–1272 (2014)] and theoretical results [*J. Am. Chem. Soc.***141**, 3110–3115 (2019), *J. Phys. Chem. Lett.* **6**, 3098–3103 (2015)].

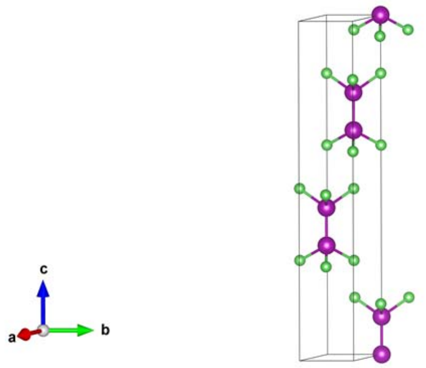

Figure R3. Schematic diagram of InSe unit cell structure, which obtained from Inorganic Crystal Structure Database (ICSD). Purple and green balls represent indium and selenium atoms, respectively. Lattices in three dimensions generally have three lattice constants (a, b, c), and angles between the sides given by (α, the angle between b and c; β, the angle between a and c; γ, the angle between a and b).

According to your suggestion, we have added this relaxed lattice constants data in the revised Supplementary Materials (page 2, line 46).

3-Band gap energy strongly depends on any semiconductor film thickness. Please discuss the band gap energy variation with the InSe layer thickness.

Response:

We thank the reviewer for the valuable suggestions. Indeed, like other two-dimensional materials, the band gap of InSe is dependent on the number of layers as well. As the number of InSe layers increases, the band gap gradually decreases from bulk value of 1.2 eV to monolayer value of 2.39 eV [*Nanoscale* **10**, 7991-7998 (2018)]. Our previous work [*Nat. Nanotechnol.* **12**, 223–227 (2017)] also reveals that the bandgap increases by about 0.5 eV with decreasing the thickness from bulk to bilayer InSe using photoluminescence spectroscopy.

However, despite the narrower band gap of thick InSe with better sunlight absorption, the devices based on thick InSe demonstrate a smaller photocurrent compare to that of monolayers. This suggests that the band gap variation between bulk and thin InSe has little contribution to the outstanding PEC performance of monolayer InSe.

We have added the above discussion in the revised manuscript (page 8, line 208).

4- Please discuss the interaction and sticking property between Ti and Quartz substrate (two different in nature, one is metal and other is insulator as glass).

Response:

We appreciate the reviewer for the suggestion. Referring to the literature [*J. Vac. Sci. Technol. A* **5,** 2890-2893 (1987)], adhesion between Ti film deposited by e-beam evaporation and quartz substrate was measured by the peel test, which value is higher than that of Ti with fused silica substrate but lower than sapphire substrate. Also, the in-situ x-ray photoelectron spectroscopy (XPS) measurements detected 2p peaks of Ti on oxide substrate, demonstrating the formation of chemical bond between Ti and oxide substrate.

We think the good sticking property between Ti and Quartz is critical for the stability of our samples. Following the reviewer's comments, we have added the above discussion in page 12, line 233 in Supplementary Materials: "Note the good sticking property between Ti and quartz also promotes the good stability of the photoanode[20]."

5-Please discuss the nature (direct/indirect) of InSe band gap energy with the deposited InSe layer thicknesses.

Response:

We thank the reviewer for the valuable suggestions. From literature, photoluminescence spectroscopy measurements show that bulk InSe is a direct band gap semiconductor, while a direct-to-indirect band gap crossover occurs when the thickness of InSe decreased down to 6 nm [*Adv. Mater.* **25,** 5714-5718 (2013)]. Theoretical calculations also showed monolayer InSe is an indirect band gap semiconductor [*Nanoscale Res. Lett* **14,** 322:1-8 (2019)]. However, although monolayer InSe with indirect band gap is not as beneficial to photoelectric conversion as bulk crystals, the former one still showed the highest PEC performance. This indicates that the nature (direct/indirect) of InSe band gap energy have negligible influence to the results of the experiment.

Accordingly, the above discussion has been added to the revised manuscript page 8, line 210.

6-It is well known that there exists a strong hybridization between Ti and InSe. There is no any discussion on this concept. Please discuss this issue with appropriate updated references.

Response:

Thanks for pointing out the critical question. According to the reports by Kistanov *et. al.* [*Phys. Chem. Chem. Phys.* **20**, 12939-12947 (2018)], there exists a strong hybridization between Ti and InSe. The Ti layer underneath will introduce an effective *n*-doping of InSe. Since the InSe crystal we used is a *n*-type semiconductor, the increase of donor atoms concentration can increase the concentration of conduction band electrons, facilitating the electron transport in InSe.

In our case, we refer to Fig. R2 that the PEC performance of InSe/Gr using different substrates have little difference between the *J*-*t* dependent characteristics, despite the different interactions between graphite, Au, Cu and Ti substrates to InSe/Gr. Therefore, the strong hybridization between Ti and InSe is not the main influencing factors of PEC performance in our system.

To clarify this point, we added the above discussion in the revised manuscript page 9, line 226 and updated the reference list: 44.

7-In Page 6, line 146, (CPE) should be introduced for the first time. It was introduced in supporting

document. It should be written as" Constant Phase Element (CPE) in the main text as well"

Response: Thanks for pointing out this point. Page 6, line 158 has been revised in manuscript as follows: "The first semicircle closer to origin represents the interface capacitance between InSe/Gr and Ti Constant Phase Element (CPE) and the second semicircle represents the interface capacitance between InSe/Gr and PEC solution."

8-In some references, the last page number of articles is missing. To be consistent with the entire manuscript, please add last page.

Response:

We thank the reviewer for the helpful comments and fully accept with this criticism. We have carefully checked the references and corrected them in the revised version.

9-There is only one reference published in 2020, please update and add some new relevant references

Response:

Thanks for the helpful suggestion. Following the comment, we update and add some new relevant references in the revised manuscript as following:

4. Takata, T. *et al.* Photocatalytic water splitting with a quantum efficiency of almost unity. *Nature* **581,** 411-414 (2020).
5. Wang, H. *et al.* Highly active deficient ternary sulfide photoanode for photoelectrochemical water splitting. *Nat. Commun.* **11,** 3078:1-11 (2020).
6. Wang, W., Xu, M., Xu, X., Zhou, W., Shao, Z. Perovskite Oxide Based Electrodes for High-Performance Photoelectrochemical Water Splitting. *Angew. Chem. Int. Ed.* **59,** 136-152 (2020).
16. Tartakovskii, A. Excitons in 2D heterostructures. *Nat. Rev. Phys.* **2,** 8-9 (2020).
17. Lyu, J., Pei, J., Guo, Y., Gong, J., Li, H. A New Opportunity for 2D van der Waals Heterostructures: Making Steep-Slope Transistors. *Adv. Mater.* **32,** 1906000:1-8 (2020).
18. Si, K. *et al.* A two-dimensional MoS_2/WSe_2 van der Waals heterostructure for enhanced photoelectric performance. *Appl. Surf. Sci.* **507,** 145082:1-11 (2020).
47. Sun, P. Z., *et al.* Limits on gas impermeability of graphene. *Nature* **579,** 229-232 (2020).

Reply to Reviewer #2

Authors provided an insight of strong coupling between surface adsorbed ions and photogenerated holes, which is critical for achieving highly efficient PEC water splitting. The results demonstrated a versatile method of stabilizing chemical sensitive electrodes materials and emphasized the importance of choosing specific 2D electrodes to tune ions concentration near electrode surface. The manuscript is well organized and conclusions are well supported on calculation results. I recommend the publication of the present work after revisions.

Response:

We thank the Reviewer for careful reading of our manuscript and helpful comments.

1. The author should explain the difference in scale between experiment and simulation.

Response:

We thank the reviewer for the valuable suggestions. In our simulations, for InSe/Gr, the box is 36.9 × 36.9 × 30 $Å^3$, which contains 774 atoms. And for MoS_2/Gr, the box is 29.52 × 29.52 × 25 $Å^3$, with a total of 531 atoms. The convergence criterion is that when the energy change at each atom is less than 0.1 eV ($\Delta E <$ 0.1 eV per atom).

Although the simulation scale (nanometers) is smaller than the real sample scale (micrometers), we note that the crystals have periodic lattice structures. Considering surface periodicities of (15 ×15) and (9 × 9) for graphene and InSe, respectively, the in-plane lattice constant of the hybrid structures was adjusted to the lattice constant of the graphene supercell, and the lattice mismatch (δ) at the interface was calculated as 1.06 % using equation [*Chem. Rev.* **120**, 2123–2170 (2020)]:

$$\delta = \frac{a_g - a_{semi}}{a_g} \times 100\% \qquad (\text{R1})$$

where a_g and a_{semi} are the original lattice constants of the graphene and semiconductor material, respectively.

Using the same method, the mismatch strain in MoS_2 layer was 3.45% (please see the details in Table R1). In fact, such a negligible effect of the small lattice mismatch on the electronic structure has been reported in other graphene-based hybrid systems simulation [*Nature* **567,** 323-333 (2019), *Phys. Chem.*

Chem. Phys., **20,** 12939–12947 (2018)]. Therefore, we believe that this scale is reasonable for simulating our experiment systems. Accordingly, we have added the above discussion in the revised Supplementary Materials (page 2, line 37).

Table R1. Lattice constants and lattice mismatch of the graphene and semiconductor material.

System	Graphene	InSe	Graphene	MoS_2
Primary(Å)	2.46	4.056	2.46	3.167
Super cell	15 x 15 x 1	9 x 9 x 1	12 x 12 x 1	9 x 9 x 1
Number of atoms	450	324	288	243
Lattice a(Å)	36.9	36.51	29.52	28.50
Mismatch		1.06%		3.45%

How to make the results of experiment and simulation confirm each other? How to prove that the simulation structure is consistent with the experimental structure?

Response:

Thanks for the valuable comment. To make the results of experiment and simulation confirm each other, there are a few points need to be confirmed: 1) whether the simulation structure is consistent with the experimental; 2) if the PEC measurement condition is reproduced in simulations; and 3) whether the simulation results support the experimental findings.

For the first point, to ensure the simulation structure is consistent with the experimental structure, we used fabrication methods that were reported to best keep the perfect crystal structure and quality of the single 2D crystals:

a) The crystals are mechanically exfoliated in the glovebox filled with inert gas (Argon) atmosphere, which can make sure the crystals contain virtually little, if any, surface defects. [*Solid State Commun.* **146,** 351-355 (2008), *Nano Lett.* **11,** 2396–2399 (2011)]. We updated the reference list: 35,36.

b) We used the dry peel transfer technique for heterostructure assembly following the method in our previous work to keep the interface atomically clean [*Nat. Nanotechnol*. **12**, 223-227 (2017) and *Nano Lett.* **15**, 4914–4921 (2015)], without any contact of water and organic solvent. As a result, our devices are extremely stable under ambient conditions for months even after multiple PEC measurements as shown in Supplementary Fig. 7.

To further prove the validity of our simulation model, as discussed in Supplementary Materials page 3, line 70, we calculated the interlayer distance between InSe/Gr ($d_{InSe/G}$) and MoS_2/Gr ($d_{MoS2/Gr}$) heterostructures and compare the results to that reported in literature. The values are 3.44 Å for $d_{InSe/Gr}$ and 3.36 Å for $d_{MoS2/Gr}$ respectively, in good agreement with the values of 3.3-3.4 Å reported from both theoretical calculation and cross-section characterization of typical graphene and transition metal dichalcogenide (TMDC) interlayer distance [*Phys. Rev. B* **93**, 155104:1-10 (2016), *Science* **353**, aac9439:1-11 (2016) and *Nat. Nanotechnol.* **10**, 534–540 (2015)].

For the second point, the solution in our PEC test system contains only sodium hydroxide, so it is reasonable to only consider the effect of OH^- near the heterostructure surface in simulations.

For the third point, as demonstrated in the main text, using our model, the difference of predicted energy change before and after OH^- adsorption between MoS_2/Gr and InSe/Gr is close to that estimated using experimental data, and well describes the experimentally observed higher photocurrent from InSe/Gr structures (please see the main text page 9, line 240 and Supplementary Materials page 4, line 101). Therefore, we believe that our simulation and experiment confirm each other.

2.The exfoliation energies of InSe monolayer should be mentioned in Supplementary Materials (Refer to Energy Storage Materials 25 (2020) 866–875)

Response:

We thank the reviewer for the helpful suggestion. The n-layer InSe exfoliation energy per unit area $E_{exf}(n)$ can be calculated by [*Energy Storage Materials* **25,** 866–875 (2020)]:

$$E_{exf}(n)= \frac{E_{iso}(n) - \frac{n}{m}E_{bulk}}{A} \qquad (R2)$$

Where $E_{iso}(n)$ is the energy of the unit cell of an isolated n-layer slab in vacuum, E_{bulk} is the energy of a bulk material with m layers, and A is the in-plane area of the bulk unit cell.

Using the corresponding values as shown in Table R1, the exfoliation energy of InSe we calculated is 14.5 meV A^{-2}, which is agree with the reported binding energy of 14.9 meV A^{-2} for single layer InSe [*Nat. Nanotechnol.* **13**, 246–252 (2018)].

According to the reviewer's advice, we added the above discussion and Table R2 on page 2, line 51 in the revised Supplementary Materials, and updated the reference list: 10.

Table R2. Exfoliation energy of monolayer InSe.

System	E_{iso} (eV)	E_{bulk} (eV)	A (Å^2)	n	m	E_{exf} (meV A^{-2})
InSe	-14.797	-45.109	16.456	1	3	14.5

3.In Fig.1c, 4. how to determine the adsorption position of hydroxide ions on the InSe/Gr heterostructure surface?

Response:

Thanks for the valuable comment. From the initial structure of hydroxide ions near the InSe/Gr and MoS_2/Gr anode as shown in Supplementary Fig. 4, after the optimization by CP2K, a most stable structure can be obtained. As the final adsorption configuration shown in Fig. 1c, the hydroxide ions adsorb on top of the carbon atom of graphene, which is consistent with other report [*J. Phys. Chem. Lett.* **7**, 4695–4700 (2016)].

Is the process of multiple hydroxide ions considered?

Response:

Thank you for the comment. Our simulation was mainly focused on the mechanism for the different PEC performance between InSe/Gr and MoS_2/Gr. Although the coverage of multiple hydroxide ions may cause interactions between hydroxide ions, the difference in the energy change of hydroxide ions on InSe/Gr and MoS_2/Gr will not change. Thus here we did not consider the process of multiple hydroxide ions.

Does the author consider the change of free energy in the process of water splitting?

Response:

Thanks for the suggestion. In our alkaline system, we mainly focused on the reaction of hydroxide ions with holes on the electrodes surface, which is usually considered as the first transition state of water splitting: $OH^- + h^+ \rightarrow OH^*$. Accordingly, we have optimized the simulation system and recalculated the energy change between the initial and transition state, as shown in Supplementary Table 4 in the revised manuscript. The free energy changes (ΔE) on InSe/Gr and MoS_2/Gr electrodes are 0.462 eV and 0.579 eV, respectively. The smaller ΔE on InSe/Gr and the ΔE difference of ~ 0.117 eV between the two systems are in good agreement with our experiment results. Following the reviewer's comment, we added the optimized simulation results in the revised manuscript page 9, line 240 and in page 4, line 101 of the

revised Supplementary Materials.

4. Why do authors choose to discuss the situation when pH = 13?

Response:

Thank you very much for the comments. pH = 13 is a critical experimental condition in our manuscript. Thus we also use pH = 13 in our simulations to keep the same condition with experiments.

The reason for the measurements at pH= 13 is as follows: Firstly, catalytic studies have demonstrated that many materials are able to offer very good OER performance under alkaline conditions. [*Nat. Energy* **4**, 519–525 (2019)*, J. Mater. Chem. A* **5**, 11634-11643 (2017)]. At present, most of the photoanode materials such as TiO_2 [*Energy Environ. Sci.* **8**, 2825-2850 (2015)] and Fe_2O_3 [*Nat. Commun.* **10**, 5599:1-10 (2019)] are tested in alkaline solutions. Other materials such as ZnO [*Nat. Commun.* **10**, 4875:1-10 (2019)] and $BiVO_4$ [*Nat. Commun.* **10,** 2609:1-9 (2019)] are tested in acidic or neutral solutions, mainly because they cannot stably exist in alkaline. InSe/Gr photoanode can keep stable in alkaline conditions, so we choose to do the PEC tests in alkaline conditions.

Secondly, as shown in Fig. 2a, at low NaOH concentrations C, the J_{max} is governed by surface charge. To minimize the contribution of the surface charge induced hydroxide ions accumulation, we choose high C (pH=13) as the working condition in most of the PEC measurements.

Reply to Reviewer #3

The authors present in this article the deposition, analysis and application of InSe crystals protected by graphene. The photocurrent densities obtained are sound and they present useful and interesting characterization.

Response:

We are grateful for this kind assessment.

However, the work does not seem complete enough and in the typical format and detail of Nature Communications. For example

-There is no paragraph introducing the paper at the end of the introduction.

Response:

We thank the reviewer for the helpful comments and fully accept with this criticism. According to the comment, we add the following paragraph in the revised manuscript (page 3, line 65) :

With this in mind, here we present a new class of photoanode made from single crystal InSe encapsulated with monolayer graphene with schematics as shown in Fig. 1a. The unique feature detailed herein is the atomic-scale thickness of 2D InSe/graphene (InSe/Gr) photoelectrodes, allowing us to investigate the surface effect to PEC performance. The encapsulation by graphene offers a reliable method to enhance the stability of InSe anode down to monolayer during long time PEC cell operation. Using the 2D heterostructure based photoanode, we observed an ultrahigh photocurrent density > 10 mA cm^{-2} at 1.23 V versus reversible hydrogen electrode (RHE). Detailed investigation of both experiments and DFT simulations reveal the importance of hydroxide ions interaction with trapped photo-generated holes near the anode surface. As a result, we also found a persistent current even after illumination off for thin layers of InSe/graphene photoanode, which was not observed on other 2D photoelectrodes.

- There is no discussion.

Response:

We thank the referee for his/her insightful comments that helped us to make the paper clearer and

stronger. Accordingly, we have modified the discussion and conclusion paragraph in the revised manuscript page 9, line 217, further investigating the origin of the high performance from InSe/Gr photoanodes.

-The Experimental does not have many details, especially in the deposition of the films (could this paper be reproduced by a PhD student?)

Response:

Thanks for pointing out the critical question. Following the comment, we have modified the “Photoanode fabrication” part in Methods (page 11, line 274), adding more details about the assembly of 2D heterostructure and the deposition of the film as follows.

Photoanode fabrication. Schematic diagram of InSe/Gr anode fabrication process as shown in Supplementary Fig. 12. To avoid the degradation of ultrathin InSe during the following measurements, the exfoliation and encapsulation of the InSe photoanodes were carried out in an inert atmosphere provided by a glovebox (H_2O and O_2 below 0.1 ppm). Firstly, InSe flakes were mechanically exfoliated onto a thin polypropylene carbonate (PPC) film. Then, a monolayer InSe crystal was identified under the optical microscope and was lifted with monolayer graphene (Gr) attached on polymethyl methacrylate (PMMA) membranes using dry peel transfer technique[21,25]. The resulting Gr–InSe stack was transferred onto a 30 nm Ti electrode prepatterned by photolithography and e-beam evaporation with quartz substrate. The obtained heterostructures were heated at 70 ºC for 1 h and then the PMMA was dissolved in acetone and isopropanol. Since the top single layer graphene is impermeable to all gas and liquid molecules[47], such encapsulation can protect the ultrathin InSe during the following PEC measurements and keep its surfaces away from other contamination. Finally, the sample was taken out of the glovebox to verify the thickness of InSe using atomic force microscope. Similar fabrication procedures were used for the MoS_2/Gr anodes.

In terms of reproducibility, we note that following our fabrication procedures as described above, similar samples have also been successfully fabricated in Prof. Ye Yu’s lab in Peking University and tested in the lab of Prof. Guo Yuxi and Prof. Lin Zhan in Guangdong University of Technology. We have measured 35 samples in total by 3 different students, all samples showed reproducible performance.

So we believe this method and experiment results could be reproduced by other PhD student.

Are there more crystals in the electrode contributing to the photocurrent?

Response:

In the process of the InSe-Gr heterostructure assembly, we carefully transfer the 2D flakes onto Ti substrate, making sure that the InSe/Gr heterostructure is the only crystals on the electrode, as shown in Fig. R4.

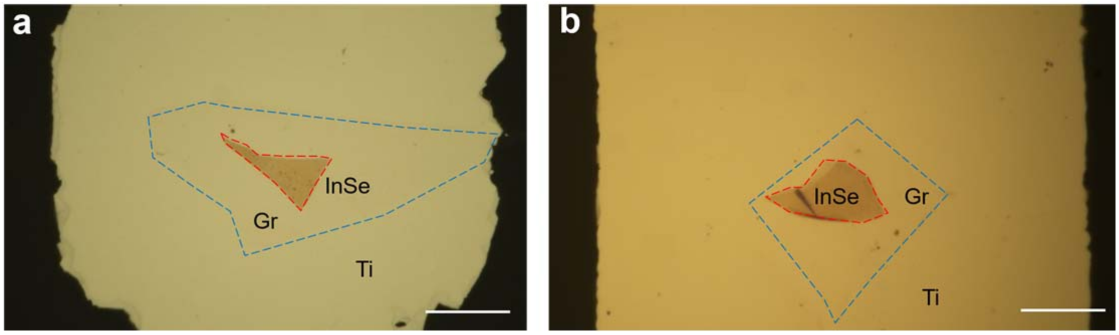

Figure R4. Optical images of monolayer InSe/Gr heterostructures on metal electrodes. The blue and red contours indicate the area of monolayer graphene and InSe, respectively. The scale bars are 20 μm.

How is the area calculated?

Response:

The area used to calculate the current density is the area of the InSe flakes which is evaluated by the software NIS-Elements D with Nikon ECLIPSE LV100ND optical microscope after we outline the flake profile in the optical image.

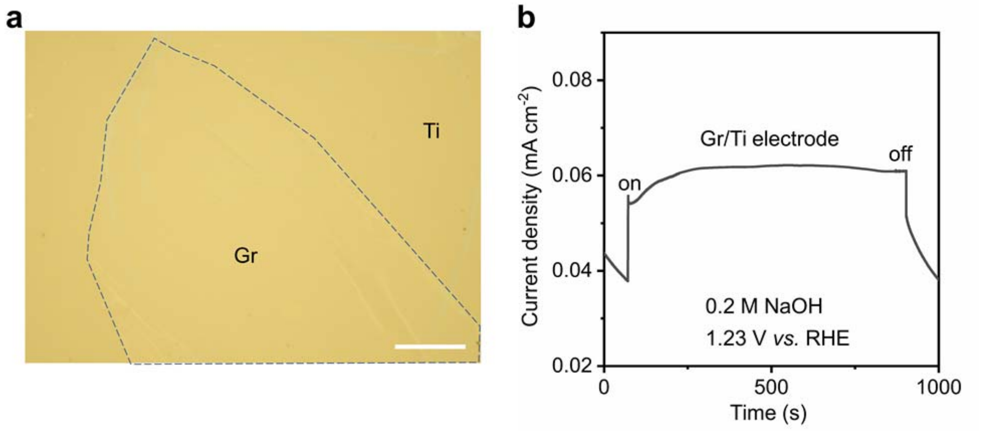

Figure R5. PEC measurements and optical image of monolayer graphene. **a** Optical image of monolayer Gr on Ti electrode, the blue contours indicate the area of monolayer graphene. The scale bar is 20 μm. **b**

Photo current density of graphene/Ti electrode as a function of time when light on or off. J_{max} is about 0.024 mA cm^{-2}.

To investigate the current contribution from the area of graphene encapsulation layer, in the control device composed of only graphene and Ti electrode without InSe (shown in Fig. R5 and Supplementary Fig. 6 in the revised Supplementary Materials), J_{max} is found close to 0.024 mA cm^{-2}, three orders of magnitudes lower than that from InSe/Gr photoanode. It suggests that the InSe crystals play the key role to the ultrahigh photocurrent and thus it is reasonable to take the area of InSe flakes as the effective area of the PEC device.

- Some wording could be improved or corrected (wrong use of "logit", "virgin curves" better simply called "linear-scale curves" ...)

Response:

We thank the reviewer for pointing out the typos in our manuscript and have corrected them in the revised version as following:

"The original linear-scale curves is shown in Supplementary Fig. 5." in page 5 line 126.

"The original linear-scale curves of the one in Fig. 1d:" in Supplementary Materials page 10 line 213.

- Fig 3c would be read better in a 2D graph

Response: We appreciate the helpful suggestion and have modified the Fig 3c as below.

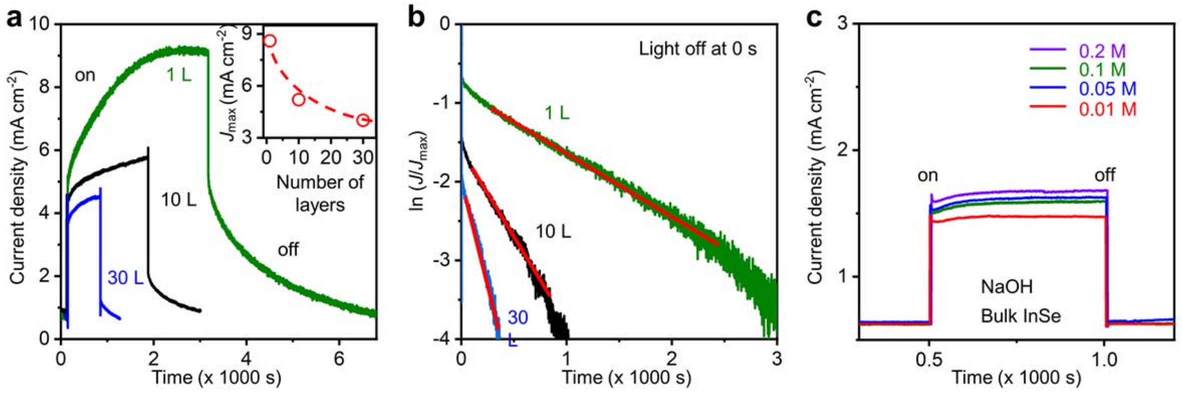

Reviewer #2 (Remarks to the Author):

This manuscrpt should be accepted.

Responses to the reviewers' comments:

Reply to Reviewer #2

This manuscrpt should be accepted.

Response:

Thank you very much for your positive comments.
